# Supplementary material for: DeepETPicker: Fast and accurate 3D particle picking for cryo-electron tomography using weakly supervised deep learning
Source: Nat Commun. 2024 Mar 7;15:2090. doi: 10.1038/s41467-024-46041-0 (PMC11258139; doi:10.1038/s41467-024-46041-0)
Supplement: Supplementary file 1 — Supplementary Information [file 41467_2024_46041_MOESM1_ESM.pdf]

Supplementary Information

DeepETPicker: Fast and accurate 3D particle picking for cryo-electron tomography using weakly supervised deep learning

Guole Liu*, Tongxin Niu*, Mengxuan Qiu, Yun Zhu, Fei Sun^, and Ge Yang^

A. Supplementary Methods

A.1 Preprocessing of Tomograms

A zero-mean normalization of voxel values is carried out for each input tomogram as follows:

$$x^* = \frac{x - \mu}{\sigma} \tag{1}$$

where $x \in \mathbb{R}^{N\times N\times N}$ denotes the input tomogram, μ and σ denote the mean and standard deviation of x, respectively, and x^* denotes the zero-mean normalized tomogram.

A.2 Software Design

DeepETPicker is open-source software implemented in Python with a user-friendly graphical interface (Supplementary Fig. 1). It integrates multiple functions, including picking of particles, visualization of annotated particles, pre-processing of input tomograms, generation of simplified masks, and configuration of parameters for training and inference. DeepETPicker picks 3D particles of varying sizes and structures from simulated and experimental cryo-ET datasets with the best overall speed and accuracy in comparing with competing state-of-the-art methods.

A.3 Hyperparameter Setting

There are several hyperparameters (t_g, t_{seg}, t_{dist}, .etc) for DeepETPicker. Here, we discuss how the setting of these hyperparameters affects results of particle picking.

$$M = \{(x, y, z) \mid x, y, z \in [-r, r] \cap Z\} \tag{2}$$

$$mask_{tball}(x, y, z)|_{(x,y,z)\in M} = \begin{cases} c & if\ e^{-\frac{x^2+y^2+z^2}{2r^2}} > t_g \\ 0 & otherwise \end{cases} \tag{3}$$

According to Equations (2, 3), the hyperparameter t_g determines the shape of Truncated-Ball masks ($mask_{tball}$). The mask of $mask_{tball}$ becomes a Ball mask when $t_g \geq \exp(-0.5) \approx 0.607$, and it becomes a Cubic mask when $t_g \leq \exp(-1.5) \approx 0.223$. Therefore, $mask_{tball}$ will be a Truncated-Ball mask when $t_g \in (0.223, 0.607)$. When t_g approaches

0.223, the generated Truncated-Ball mask would be very similar to a Cubic mask; When t_g approaches 0.607, the generated Truncated-Ball mask would be very similar to a Ball mask. To generate a Truncated-Ball mask that differs sufficiently from Ball/Cubic masks, we choose the middle point between -0.5 and -1.5 and set $t_g = \exp(-1) \approx 0.368$ in this study. When $t_g = 0.368$, equation (3) can be simply described as follows:

$$mask_{tball}(x,y,z)|_{(x,y,z)\in M} = \begin{cases} c & if\ \sqrt{x^2+y^2+z^2} < \sqrt{2}\,r \\ 0 & otherwise \end{cases} \tag{4}$$

We initially used Ball masks (Ball-M) as our weak labels and tested different radius settings. However, the experimental results showed that Ball-M with a diameter of for example 7 usually could not pick all types of particles. Often one or more types of particles were missed. This motivated us to examine weak labels with different shapes, which should be easy to implement and should have good approximations in voxels for actual particle masks. The first type of weak labels we considered were Cubic masks (Cubic-M). However, because the surfaces of macromolecular particles are usually smooth, we found the regions near the edges of Cubic-M were noisy. To reduce the number of noisy voxels, we tried a new type of weak labels, Truncated Ball masks (TBall-M), which do not have the same sharp edges as Cubic masks. Overall, we examined these three different types of weak labels under different radius settings. Compared to Cubic-M and Ball-M masks, TBall-M masks provided more stable and better localization and classification performance, regardless of what radius was chosen (Fig. 2d and Supplementary Table 3). Another more important conclusion based on our experiments was that utilizing simplified masks with constant diameters as training labels achieved comparable, if not better, performance as real segmentation masks. Furthermore, simplified masks with constant diameters avoided the issue of class imbalance and simplified the selection of loss functions (Supplementary Methods A.5). Because TBall-M masks consistently achieved good performance in particle picking, we did not investigate other more complex shapes such as polygons.

The output score maps of 3D-ResUNet are in the range of [0,1], in which the value of each voxel denotes its probability score of belonging to a certain class. t_{seg} is a selected threshold that transforms a soft score map into a binary map: a voxel with its value below t_{seg} is labeled

as 0 and otherwise as 1 so that a binary map is generated. The influence of t_{seg} on the classification performance of DeepETPicker trained by different types of masks on SHREC2021 dataset is summarized in Supplementary Table 11. The results show that t_{seg} has little effect on the classification performance when it varies from 0.1 to 0.9. Therefore, we set the default value of t_{seg} to 0.5 in this study.

The hyperparameter t_{dist} is the threshold for the minimal Euclidean distance between two particles. Normally, t_{dist} is set to be half of the diameter of the particle $\left\lceil \frac{d}{2} \right\rceil$, where $\lceil\cdot\rceil$ denotes the round-up operation. If the Euclidean distance between two particles is lower than t_{dist}, the two particles are considered the same.

The hyperparameter t_{lm} is a threshold determining whether a local maximum is a particle. In our study we set t_{lm} as a constant of 0.1. The hyperparameter N is the size of a subtomogram. It needs to be a multiple of 8. It is recommended that this value be no less than 64, and the default value is 72. The hyperparameter pad_size is the padding size for the overlap-tile strategy. Usually, it ranges from 6 to 12, and the default value is 12. The hyperparameter max_epoch is the total number of training epochs. The default value 60 is usually sufficient. The hyperparameter $batch\_size$ is the number of samples processed before the model is updated. It is determined by the GPU memory. Reducing this parameter may be helpful if an out-of-memory error is encountered.

In summary, while t_{dist} is set to be a half of the particle diameter and $batch\_size$ is determined by the GPU memory, other hyperparameters can generally use their default values. A more detailed description for the choice of hyperparameters can be found in https://github.com/cbmi-group/DeepETPicker.

A.4 Ablation Study for 3D-ResUNet Architectural Customizations

Coordinated convolution incorporates the spatial context of the input images into the convolutional filters, while image pyramid inputs preserve features of input images at different resolution levels, which can effectively improve the performance of convolutional neural networks. For validation, an ablation study for coordinated convolution and image pyramid

inputs was carried out (Supplementary Table 12). We can observe that coordinated convolution or image pyramid inputs improve the mean F1-score by ~1.5% individually. They mainly improve the classification performance of tiny particles. When coordinated convolution and image pyramid inputs are added simultaneously, the mean F1-score of all complexes improves by 4.2%, and the mean F1-score of tiny complexes improves by 8.1%. An ablation study is also carried out for channels of 3D-ResUNet. We find that 3D-ResUNet with channels of [8, 16, 24, 36] achieves comparable performance to that of [24, 48, 72, 108] (Supplementary Table 13). The model size of 3D-ResUNet with channels of [8, 16, 24, 36] is only 3.4 MBytes, which validates its architectural efficiency.

A.5 Ablation Study for Loss functions

A detailed study of different types of "weak labels" shows that simplified masks with constant diameters can be used to replace particles with different diameters, achieving performance comparable to that of real masks. Using simplified masks with constant diameters as the training labels eliminates the problem of class imbalance and simplifies our selection of loss functions. We performed an ablation study for loss functions, demonstrating that different losses achieve similar picking performances when using simplified masks with constant diameters (Supplementary Table 14).

A.6 Ablation Study for Data Augmentation

The data augmentation used in our study is composed of two types of transformations, namely mirror transformation and spatial transformation (including random cropping, elastic deformation, scaling and rotation). An ablation study was carried out for these two types of transformations (Supplementary Table 15). We find that on the SHREC2021 dataset, mirror transformation substantially improves classification F1-score by 8.3% and slightly improves the localization F1-score by 1.0%. Spatial transformation improves the localization F1-score by 2% and classification F1-score by 2%. This is because the spatial transformations such as random rotation/cropping effectively increase the diversity of the tomogram training data. When mirror transformation and spatial transformation are used jointly, the mean F1-score of classification is

improved by 9.5% and the F1-score of localization is improved by 3.4%. The ablation study described above indicates that mirror transformation can effectively improve classification performance.

A.7 Threshold settings for DeepFinder and TM

For DeepFinder, its processing of a dataset would generate a file with five columns, i.e., class label, x, y, z and cluster size. For particle coordinates of clustering results from DeepFinder, we found that the number of particles it detected is much larger than the number of manually labeled particles. When we plot the picked particles back to the tomogram, we found that there are many false-positive particles. If we use the particles detected by DeepFinder for sub-sequent analysis, the performance of DeepFinder will be underestimated. For DeepFinder, the voxels of the cryo-ET tomogram were first classified into N classes. Then the multi-class voxel-wise classification map was spatially clustered into 3D connected components, with each cluster corresponding to a unique particle. In the original paper of DeepFinder, it was written that "Clusters that are significantly smaller than the size of target particles are considered as false positives and are discarded". Thus, for a fair comparison with DeepFinder, we interactively adjusted the volume threshold as 0-20% the size of target particles based on visual inspection. Indeed, when we compared the F1-scores of DeepFinder with and without setting the volume thresholds on the testing set, we found that for the three experimental datasets the F1-score consistently improved after setting the thresholds.

- For the EMPIAR-10045 and EMPIAR-10499 datasets, the diameter of ribosomes is about 23~24 voxels. During the training stage, we use spheres with a radius of 11 as labels. For a sphere with a diameter of 23, its volume can be calculated as $V = \frac{4\pi r^3}{3} = \frac{4\pi \times 11.5^3}{3}$. In the inference stage, we interactively adjusted the volume threshold based on visual inspection. Eventually, particles with a cluster size larger than $0.1V$ are selected as the final result for EMPIAR-10045. Particles with a cluster size larger than $0.2V$ are selected as the final result for EMPIAR-10499.

- For EMPIAR-10651, we use spheres with a radius of 11 as the labels, and its volume can be calculated as $V=\frac{4\pi r^3}{3}=\frac{4\pi\times 11^3}{3}$. In the inference stage, we interactively adjusted the volume threshold based on visual inspection. Particles with a cluster size larger than $0.1V$ are selected as the final result for EMPIAR-10651.
- For EMPIAR-11125, we use spheres with a radius of 7 as the labels, and its volume can be calculated as $V=\frac{4\pi r^3}{3}=\frac{4\pi\times 7^3}{3}$. In the inference stage, we interactively adjusted the volume threshold based on visual inspection. Particles with a cluster size larger than $0.1V$ are selected as the final result for EMPIAR-11125.

When we compared the F1-scores of DeepFinder with and without setting the volume thresholds on the testing set, we found that for the three experimental datasets the F1-score consistently improved after setting the thresholds (Supplementary Table 16 and Supplementary Fig. 16). For template matching, we use mainly the template matching function "dynamo_match" of Dynamo. There are two parameters that may affect particle selection. The parameter 'cr' (cone range) defines orientations that will be looked for inside a cone. In our experiment, we use the most typical value of 360 (sampling the full sphere). The parameter 'cs '(cone sampling) determines the scanning density inside the sphere. In our experiment, we use the most typical values of 30 (sampling the full sphere). It will generate tbl-format table files, where the tenth column shows the cross-correction coefficient. For each tomogram, we obtain a plot of the cross-correlation values found on the local maxima of the cc volume with the order. The cross-correlation values of the peaks appeared in an ascending order. We check the quality of the peaks by auxiliary clicking on the curve to select one particle and then selecting certain visualization option. We click on a few particles in the kink area in the cross-correlation to roughly estimate the cross-correlation threshold. The detailed thresholds for TM for different datasets are provided in the following Supplementary Table 17.

A.8 Split of Training/Validation/Test Sets

In practice, a scheme commonly followed by structural biologists for particle picking is to manually label a small number of particles, use these particles for training the deep learning model selected for particle picking, and, finally, use the trained model to pick particles from all tomograms.

To completely eliminate the risk of overlap between training and validation particles, for all the four experimental datasets, we manually picked particles from a tomogram that differs from the tomograms used for picking training particles. Specifically, the particles used for training were kept and the particles for validating were manually picked from a different tomogram. The particles used for the training, validation, and testing of different deep-learning based methods for experimental datasets can be found in Supplementary Table 18 and Supplementary Table 19. Taking EMPIAR-10045 as example, a total of 3120 particles are manually labeled from different tomograms. For each tomogram, the coordinates of the manually picked particles are sorted in the order of z, y, and x from the smallest to the largest. For crYOLO, DeepFinder, and DeepETPicker, 135 particles from tomo0 are used for training, and 15 particles from tomo1 are used for validation. The manually labeled particles with training and validation particles excluded are used for testing. Noting that for DeepFinder, because its initial training using 106 particles fails to converge on EMPIAR-10499 dataset, we increase the number of training and validation particles to 650 and 53, respectively.

1) For the two simulation datasets from SHREC2020 and SHREC2021 Challenges, the training, validation, and test sets were well separated by following the protocols provided by the organizers. Because a relatively small training set and a large test set are used, there can be a risk of batch-effect related to the training data. Section A11 reports the experiments that test for the potential batch-effect. The results show no evidence for such batch-effect.
2) For the experimental datasets of EMPIAR-10045 and EMPIAR-10499, the training, validation, and test set were well separated. Please refer to Supplementary Table 18 and Supplementary Table 19.

3) For the experimental datasets of EMPIAR-10651 and EMPIAR-11125, the training, validation and test sets were well separated in calculating the precision-recall curves (Fig. 5b and Supplementary Fig. 10b). Please refer to Supplementary Table 18 and Supplementary Table 19**.** However, when we calculated the B-factor, global resolution, local resolution, and log-likelihood distribution for two of the experimental datasets, EMPIAR-100651 and EMPIAR-11125, we combined the training and validation particles with the testing particles for reconstruction. This was mainly because the numbers of particles were very limited. Although this does affect the reported B-factor, global resolution, local resolution, and log-likelihood distribution, it was performed in the same way for all the methods compared to ensure a fair comparison. Furthermore, it is consistent with the practice of users in real-world applications when the goal is to use the maximal number of real particles for reconstruction.

A.9 Performance Comparison Using Precision-Recall Curves

Each of the four methods (DeepETPicker, crYOLO, DeepFinder, and TM) have a confidence metric for its picked particles. For DeepETPicker and DeepFinder, the voxels of the cryo-ET tomogram are classified into N classes. The confidence of a particle is measured by the volume of voxel-wise classification map belonging to this particle. For crYOLO, each detected particle has a confidence metric provided in the result file directly. This confidence metric denotes the probability that the detected particle is an authentic particle. For TM, the confidence of a particle is measured by the cross-correlation coefficient.

For a fair comparison between different methods, we sorted the particles of each method based on its confidence metric from the highest to the lowest. Using the manual annotation as the reference, the precision and recall of particles with confidence larger than different threshold were calculated. The precision-recall curves of different methods were then plotted together for performance comparison (Figures 3b, 4b, 5b and Supplementary Fig. 10b). This would eliminate the influence of manual setting of confidence threshold on the performance comparison of different methods.

A.10 Experiments with Different Weak Labels and Multiple Training Runs on the SHREC2021 Dataset

We have conducted two new groups of experiments with multiple training runs to check whether the performance differences observed in our study between DeepETPicker and competing models are statistically significant.

In the first group of experiments, we performed 10-fold cross-validation experiments to characterize performance of DeepETPicker on the SHREC2021 dataset, which consists of 10 tomograms. For each experiment, we randomly selected 8 tomograms for training, 1 tomogram for validation data, and 1 tomogram for testing. Mean classification F1-score is used as the performance metric. In each experiment, the same random seed is used for three different simplified masks. Overall, performance of DeepETPicker varies in the experiments (Supplementary Fig. 17a), presumably because of the different settings of simulation parameters such as defocus levels, electron doses, and particle type compositions in generating these tomograms, as reported by the organizers of the challenge (Gubins *et al*, SHREC 2021: Classification in cryo-electron tomograms, Eurographics Proceedings, 2021). For example, we observed that DeepETPicker generally achieves lower classification and localization F1-scores on tomograms with lower signal-to-noise ratios.

We found no statistically significant difference between the three types of weak labels in terms of mean classification and localization F1-scores (Supplementary Fig. 18). However, compared to Ball-M and Cubic-M labels, TBall-M masks provide more stable performance (Source Data.xlsx). Specifically, in all experiments, TBall-M picked all types of particles. In 4 out of 10 experiments, the Ball-M mask failed to pick all types of particles, missing either one or more types of particles. In 1 out of 10 experiments, the Cubic-M mask failed to pick all types of particles.

The experiments in the first group provide insights into the performance of DeepETPicker. However, the results cannot be compared directly with results of those methods from the

SHREC2021 challenge. This is because the training, validation, and test sets were partitioned following the protocols provided by the organizers. To generate results that can be used to compare DeepETPicker with the methods from SHREC2021, we performed the second group of experiments in which we followed the same protocols of partitioning training/validation/test sets and performed the experiments 10 times using 10 different random seeds. Overall, we found that the variations of both localization F1-scores and mean classification F1-scoress of DeepETPicker in the experiments are small, generally on the level of 0.003~0.005 (Supplementary Fig. 17 and Supplementary Table 20). Because only a single F1-score is provided by the SHREC 2021 organizer for each method without its statistical distribution, it is not feasible to perform statistical performance comparison of DeepETPicker versus these methods, However, given the observed low level of variations in F1-scores, the observed performance difference in e.g. Supplementary Table 4, Supplementary Table 5, Supplementary Fig. 18 and Figure 2e are generally much higher than 0.003~0.005 and therefore are likely significant. We note that it is common in deep learning studies to compare performance of competing methods without using explicit statistical tests.

Compared with Ball-M and Cubic-M masks, TBall-M mask provides more consistent localization and classification performances with highest mean and lowest standard deviation (Supplementary Fig. 18 and Supplementary Table 20**).** Through rndttest2 and ranksum analysis, there is significant difference between TBall-M and Ball-M in term of classification F1-score, with P-value 0.027 for rndttest2 and 0.023 for ranksum (Supplementary Fig. 18b). Through rndttest2 analysis, there is significant different between TBall-M and Ball-M in term of localization F1-score, with P-value 0.0432 (Supplementary Fig. 18c). Besides, similar to the conclusion of 10-fold cross-validation, TBall-M mask provides more stable classification performance than Ball-M and Cubic-M masks (Source Data.xlsx). Specifically, TBall-M picks all types of particles in all experiments. In 1 out of 10 experiments, Ball-M mask does not pick all types of particles. In 2 out of 10 experiments, Cubic-M mask does not pick all types of particles.

A.11 Testing Batch-Effect on Experimental Datasets

For experimental data, when a relatively small training data and a large testing data are used, there can be a batch-effect related to the training data. To obtain a realistic estimation of the robustness of the model performance, we fixed the validation set and the test set. We then randomly sampled five training sets to obtain five testing results on EMPIAR-10045 and EMPIAR-10499 datasets. Details on partitioning the training/validation/testing sets are summarized in Supplementary Table 21. Specifically, EMPIAR-10045 dataset consists of 7 tomograms. Five training sets and one validation set are randomly sampled from tomograms labeled *tomo0* to *tomo3*. And tomograms labeled *tomo4* to *tomo6* are used for testing. EMPIAR-10499 dataset consists of 10 tomograms. Five training sets and one validation set are randomly sampled from tomograms labeled *tomo0* to *tomo5*. And tomograms labeled *tomo6* to *tomo9* are used for testing. In this way, five testing results are obtained for both EMPIAR-10045 and EMPIAR-10499.

- For the EMPIAR-10045 dataset, three methods, i.e., DeepETPicker, Deepfinder and crYOLO, were trained by the five training sets and tested on the same test set. In terms of picking performance, compared with Deepfinder and crYOLO, DeepETPicker provides more consistent localization F1-score with the highest mean and the lowest standard deviation (Supplementary Fig. 19a). In terms of inference time, the average time for DeepETPicker is 62 seconds, which is 25 times faster than Deepfinder and 2.5 times faster than crYOLO (Supplementary Fig. 19b).

- For the EMPIAR-10499 dataset, two methods, i.e., DeepETPicker and crYOLO are trained by five training sets and measured on the same test set. Because training of DeepFinder failed to converge in training, performance metrics could not be reported. In terms of picking performance, DeepETPicker provides much more consistent localization F1-score with higher mean and lower standard deviation than crYOLO (Supplementary Fig. 19c). In terms of inference time, the average time for

DeepETPicker is 108 seconds, which is comparable to 103 seconds for crYOLO (Supplementary Fig. 19d).

B. Supplementary Figures

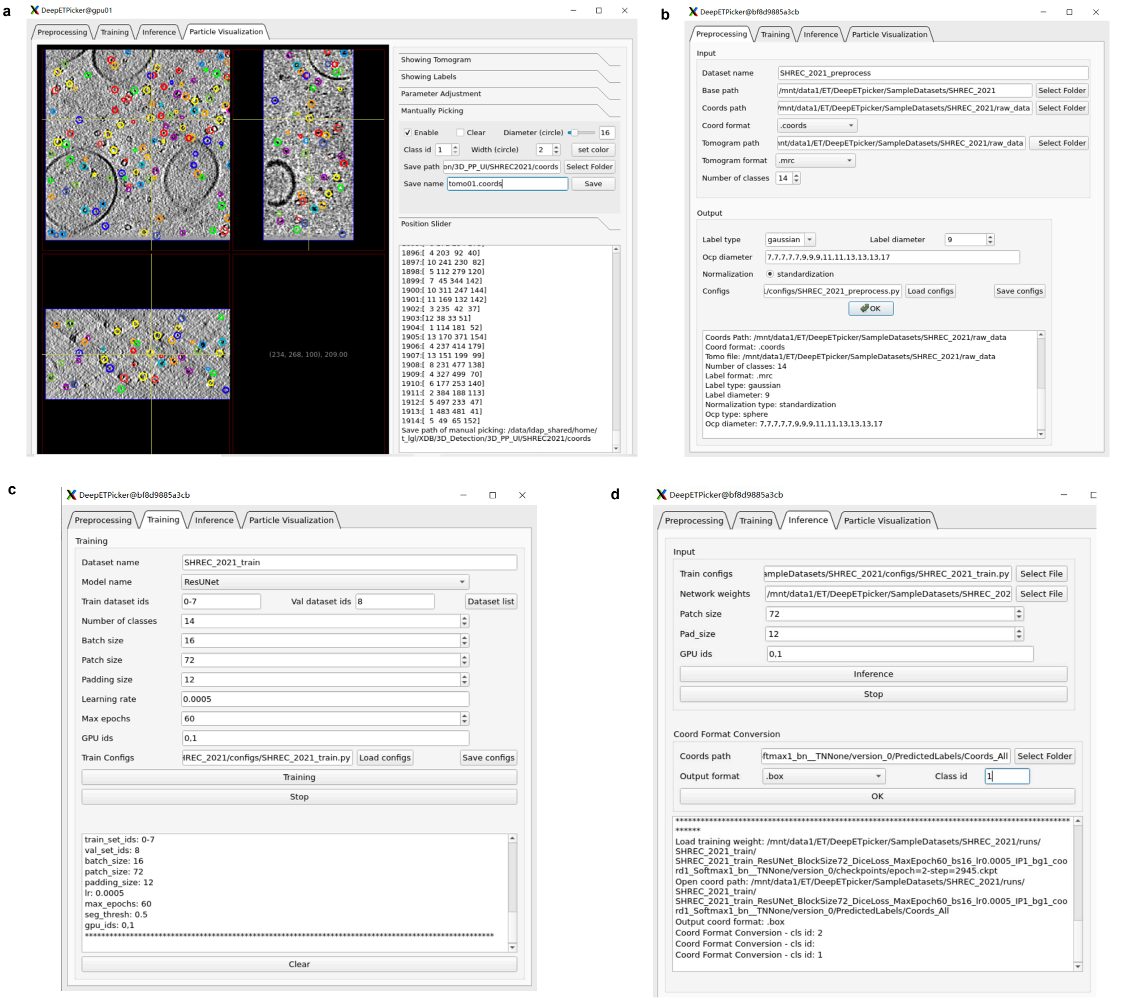

Supplementary Fig. 1 | Graphical user interface of DeepETPicker. a, The particle picking interface provides the following functions: manual annotation of particle centers and visualization of annotated particle centers. **b**, The pre-processing interface provides the following functions: pre-processing of raw cryo-ET tomograms and generation of simplified masks centered on annotated particle centers. **c**, The training interface provides the following functions: hyperparameter configuration and segmentation model selection. **d**, The inference interface provides the following functions: loading pretrained segmentation model, hyperparameter configuration, particle centers generation and format conversion for particle coordinates.

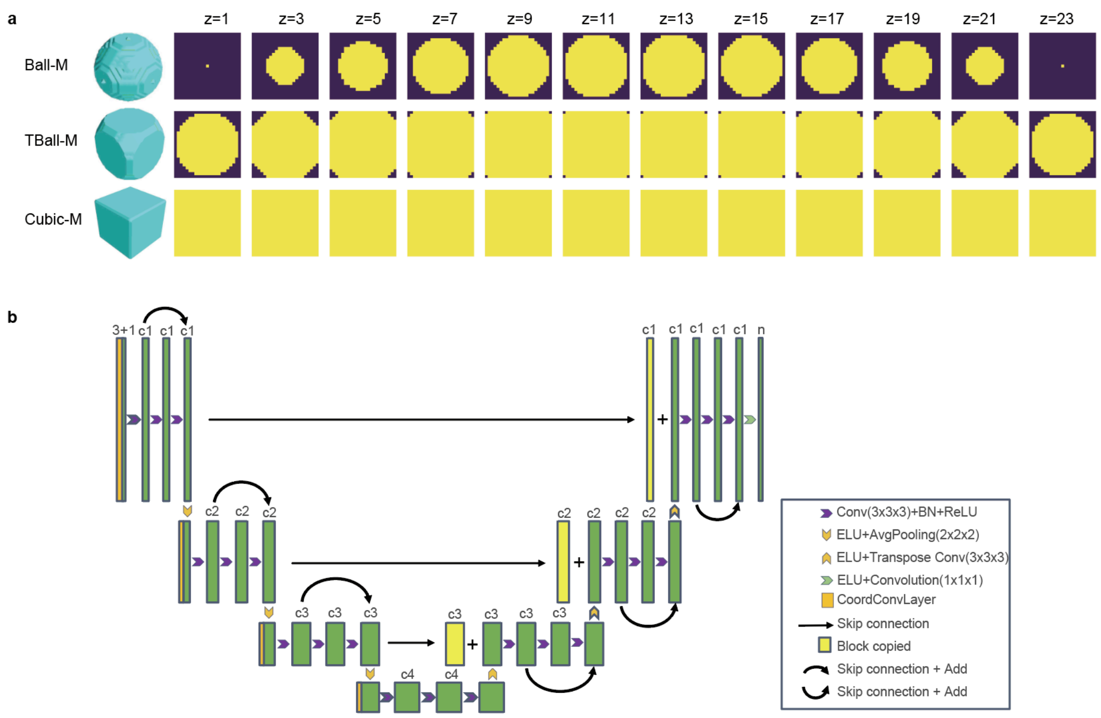

Supplementary Fig. 2 | Illustration of simplified masks and network architecture of 3D-ResUNet. a, Rendering of simplified/weak masks with a diameter $d = 23$ and their corresponding cross-sections at different positions z. Ball-M: Ball masks. TBall-M: Truncated-Ball masks. Cubic-M: Cubic masks. **b**, Detailed architecture of the segmentation neural network 3D-ResUNet, which is based on 3D-Unet[2] and adds residual connections of ResNet[3]. c1, c2, c3, c4 are feature map channels at different resolution levels.

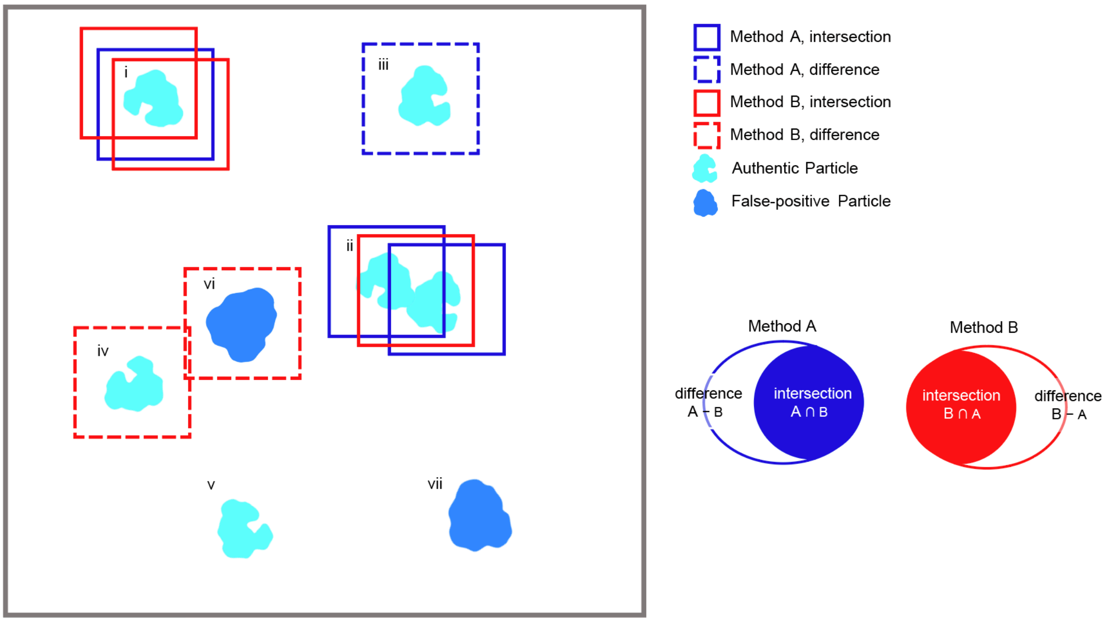

Supplementary Fig. 3 | An explanation of intersection and difference sets of particles picked by methods A and B. Solid boxes represent particles in the intersection set, and dashed boxes represent particles in the difference set. Particles picked by methods A and B are denoted in blue and red, respectively. Several representative cases of particles are shown. **Case i** shows a particle picked by both methods. Method A identifies it as a single particle, but method B identifies it as two separate particles with different centers. **Case ii** shows two particles in close proximity. Method A recognizes them as two separate particles with different centers, but method B recognizes it as a single particle. **Cases iii and iv** shows difference particles identified only by method A and method B, respectively. **Case v** shows particles missed by both methods. **Case vi** shows a false-positive particle similar in size or shape to the target particle identified by method B. **Case vii** shows a false-positive particle excluded by both methods. Either case i or ii may lead to different numbers of intersection sets between $A \cap B$ and $B \cap A$. Specifically, taking case i as an example, under the above definition of "same particles", the particle will count as 1 in $A \cap B$ and will count as 2 in $B \cap A$. In summary, the seemingly counterintuitive asymmetry is caused the above definition of "same particles". Although this counter- intuitive asymmetry can be eliminated by modifying the definition of "same particles", the current way of setting $A \cap B$ and $B \cap A$ is maintained in this study because it carries useful information.

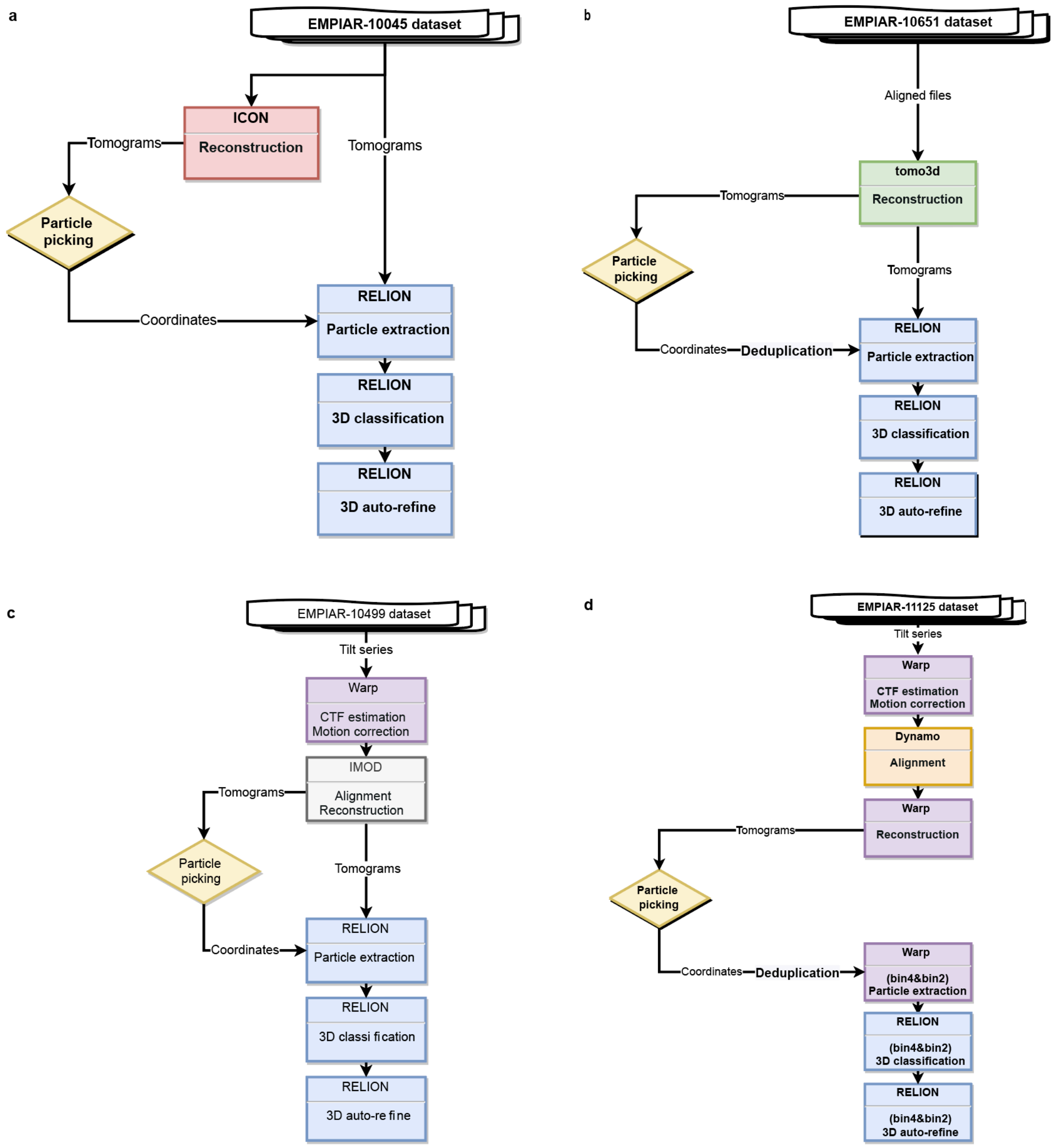

Supplementary Fig. 4 | Sub-tomogram analysis workflow. a, We use the aligned tilt series in the subdirectory of the EMPIAR entry to perform ICON reconstruction, which is then used for particle picking. The original tomograms of the entry are then utilized for sub-tomogram analysis, including CTF estimation, particle extraction, 2D classification, 3D auto-refine, and post-processing. The CTF model of each particle was generated by CTFFIND 4 in RELION 2.1.0. **b**, We use the aligned tilt series contained in subdirectory of the EMPIAR entry (EMPIAR-10651) to perform reconstruction by tomo3d (version: January 2015). Particle coordinates and the unbinned tomograms are utilized for sub-tomogram analysis by RELION 2.1.0, including CTF estimation, particle extraction, 3D classification, 3D auto-refinement. The CTF model of each particle was generated by CTFFIND 4 in RELION 2.1.0. **c**, Pre-processing operations of EMPIAR-10499 tilt series include motion

correction and CTF estimation by Warp 1.0.9, tilt series alignment by IMOD 4.9.12, and reconstruction by weighted back projection in IMOD 4.9.12. After particle picking, all subsequent processes, including particle extraction, 3D classification, 3D auto-refine, and post-processing, are performed using RELION 2.1.0. The CTF model of each particle was generated by CTFFIND 4 in RELION 2.1.0. **d**, The pre-processing of EMPIAR-11125 tilt series include motion correction and CTF estimation by Warp 1.0.9, alignment by Dynamo v1.1.509_MCR-9.6.0, and reconstruction by Warp. After particle picking, the particle coordinates are used for particle extraction in Warp and the subsequent 3D classification and auto-refinement are performed using RELION 3.1 beta.

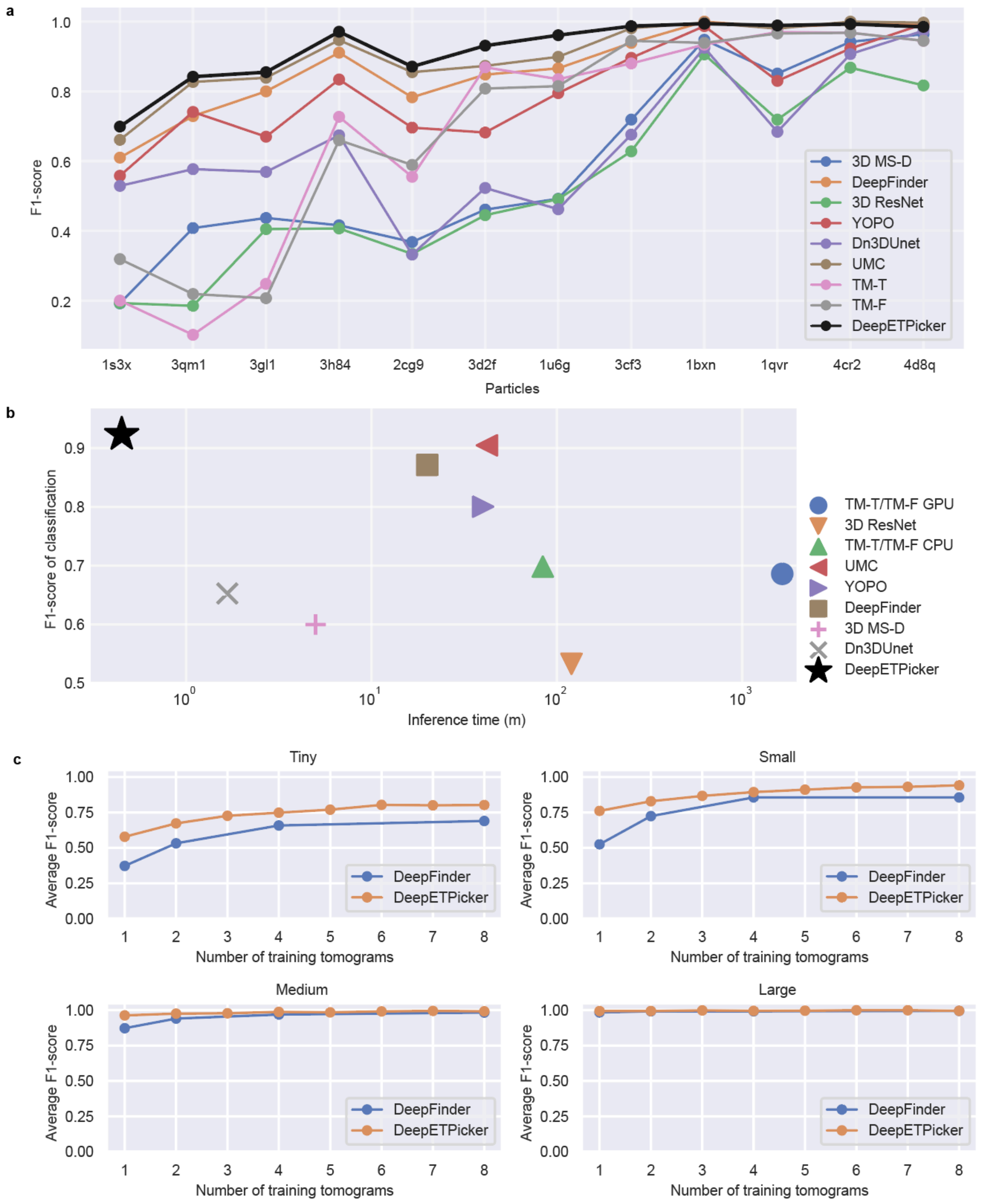

Supplementary Fig. 5 | Particle picking performance of DeepETPicker in comparison with that of competing methods on the SHREC2020 dataset. a, Classification performance measured in F1-score is plotted against particle molecular weight for DeepETPicker and other particle picking methods reported in the SHREC2020 challenge[4]. **b**, DeepETPicker runs substantially faster and achieves substantially higher classification performance than competing particle picking methods on the SHREC2020 dataset. Inference of DeepETPicker is performed on an Nvidia GeForce GTX 2080Ti. See Supplementary Table 6 for further details. **c**, Classification performance of DeepETPicker in comparison with DeepFinder measured in F1-score plotted under different numbers of training tomograms and particles with different sizes

on the SHREC2020 dataset. The particles are divided into four groups with different sizes, including Tiny (1s3x, 3qm1, 3gl1), Small (3h84, 2cg9, 3d2f, 1u6g), Medium (3cf3, 1bxn, 1qvr), and Large (4cr2, 4d8q). Source data are provided as a Source Data file.

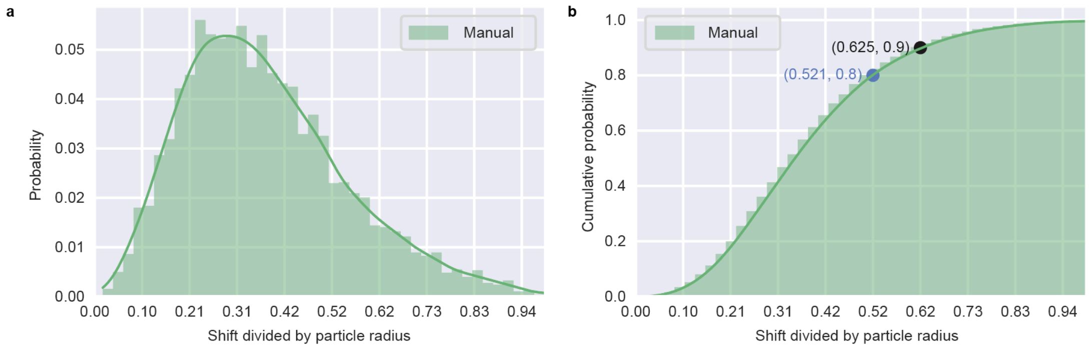

Supplementary Fig. 6 | Euclidean distances between particle coordinates obtained by manual picking and particle coordinates after refinement, based on the dataset EMPIAR-10499: Probability plot (a) and cumulative probability plot (b) of the distances, which are normalized by the particle radius.

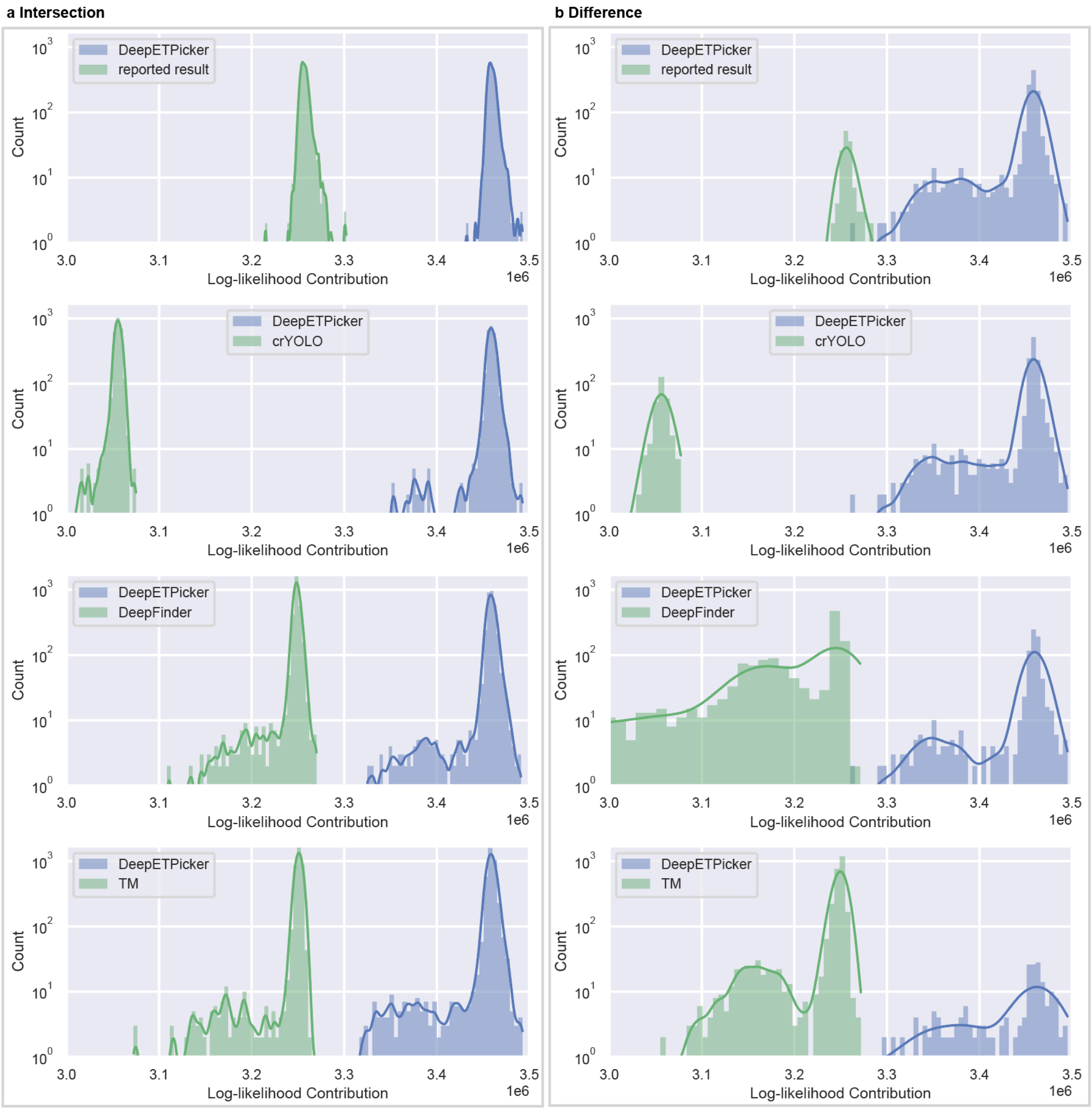

Supplementary Fig. 7 | The log-likelihood contributions of the intersection (a) and difference (b) sets of particles between DeepETPicker and the other four methods on EMPIAR-10045 dataset. The results of DeepETPicker and each of the other four methods are shown in blue and green, respectively. Source data are provided as a Source Data file.

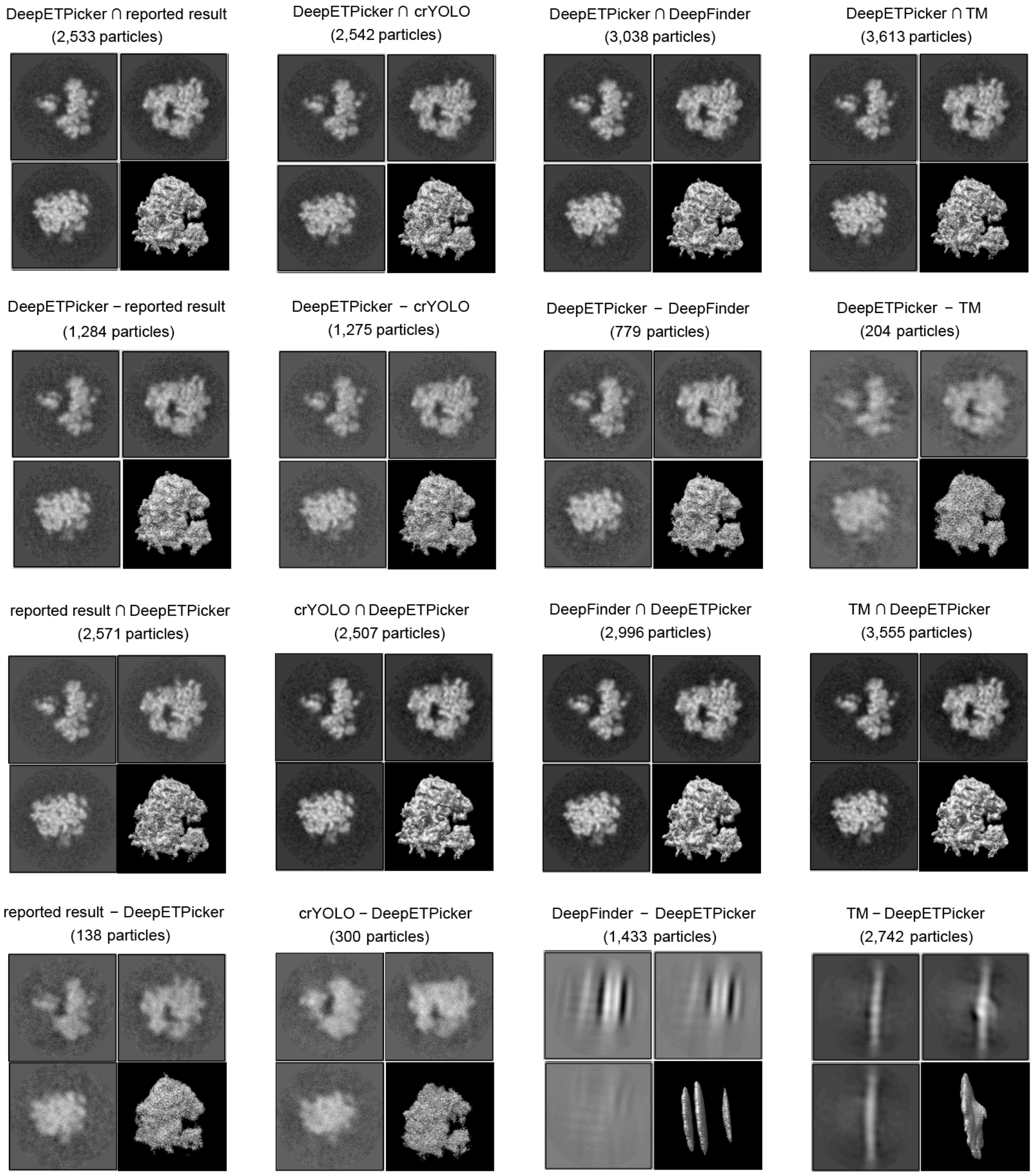

Supplementary Fig. 8 | Comparison of sub-tomogram averaging for the intersection and difference sets of particles picked by different methods on the EMPIAR-10045 dataset of *S. cerevisiae* 80S ribosome. The four figure panels are the 75th, 95th and 125th sections of the sub-tomogram averaging result and the density map. Source data are provided as a Source Data file.

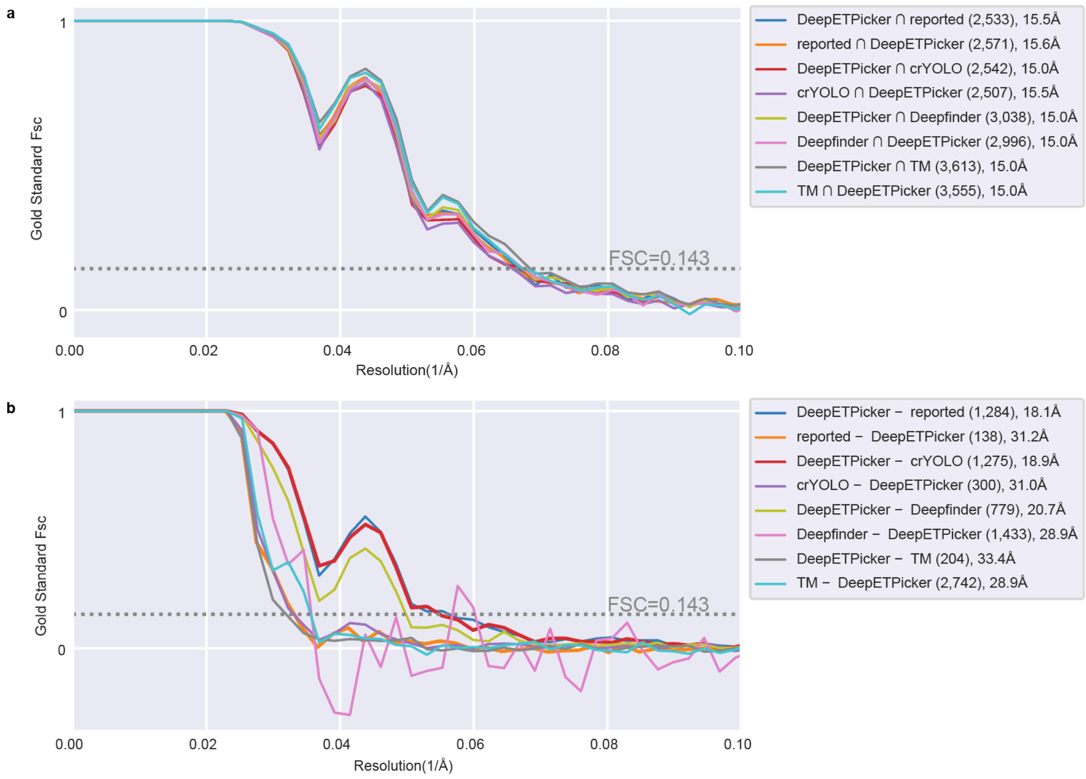

Supplementary Fig. 9 | Comparison of FSC curves achieved by the intersection (a) and difference (b) sets of particles selected by different methods on the EMPIAR-10045 dataset of *S. cerevisiae* 80S ribosome. Source data are provided as a Source Data file.

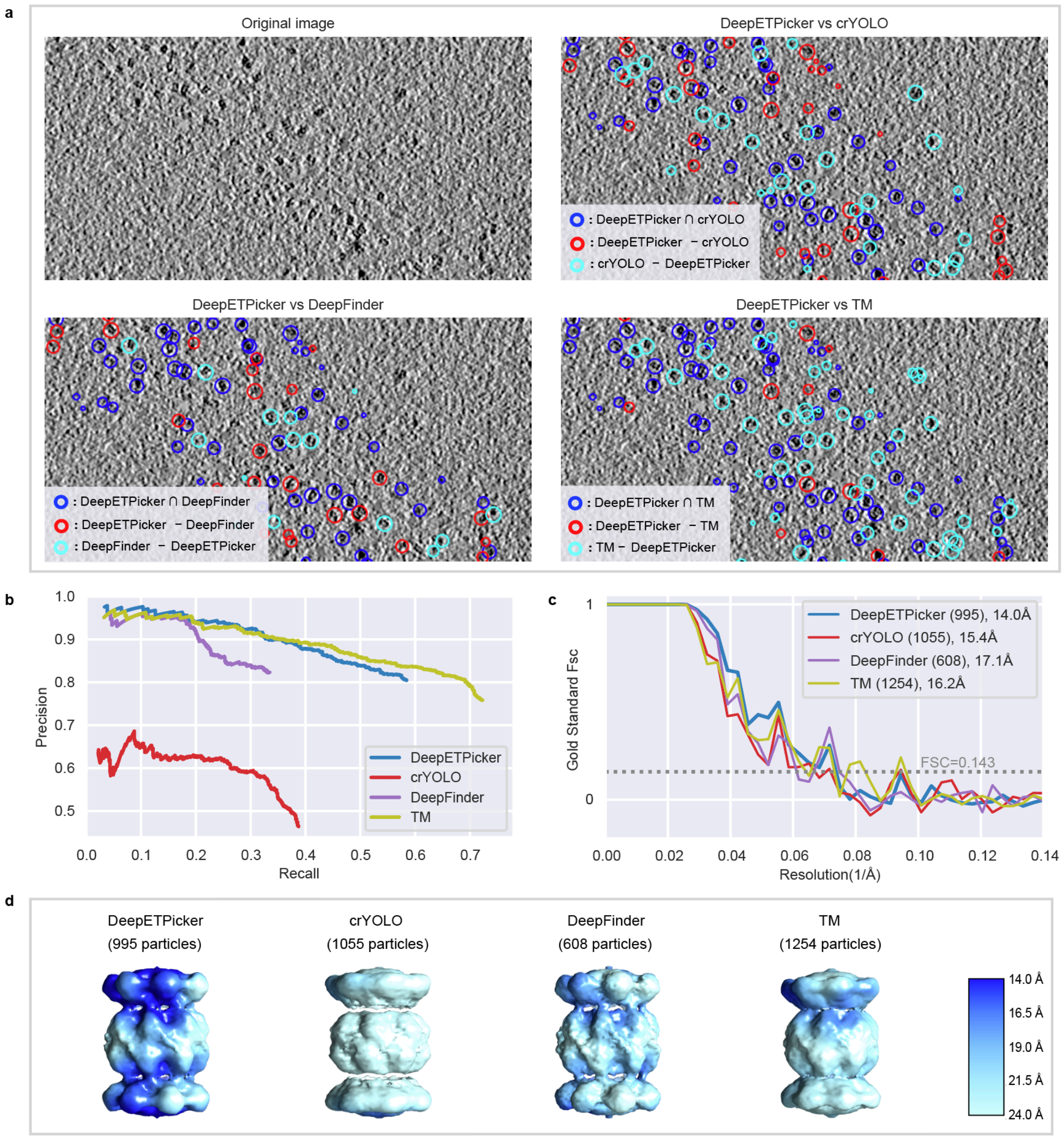

Supplementary Fig. 10 | Particle picking results on the EMPIAR-10651 dataset. **a**. Comparison of particle distributions between DeepETPicker and the other four methods (manual pick, crYOLO, template matching, and Deepfinder). Different color shows the intersection and differences of two particle sets. (This is a slice of $z = 100$ from the reconstruction of k2dft20s_14apra0023). Intersection set particles picked by DeepETPicker and the other method are shown in blue, difference set particles picked by DeepETPicker and the other method are shown in red and cyan, respectively. **b**. Precision-recall curves of different methods using manual particles as the reference. **c.** Comparative of gold standard FSC curves of particles picked by different methods on EMPIAR-10651 dataset. Of note, the oscillations of FSC curves are due to the small number of particles used. **d.** Comparison of the local resolutions of sub-tomogram averages using different particle picking

methods (DeepETPicker, crYOLO, Deepfinder and template matching). Source data are provided as a Source Data file.

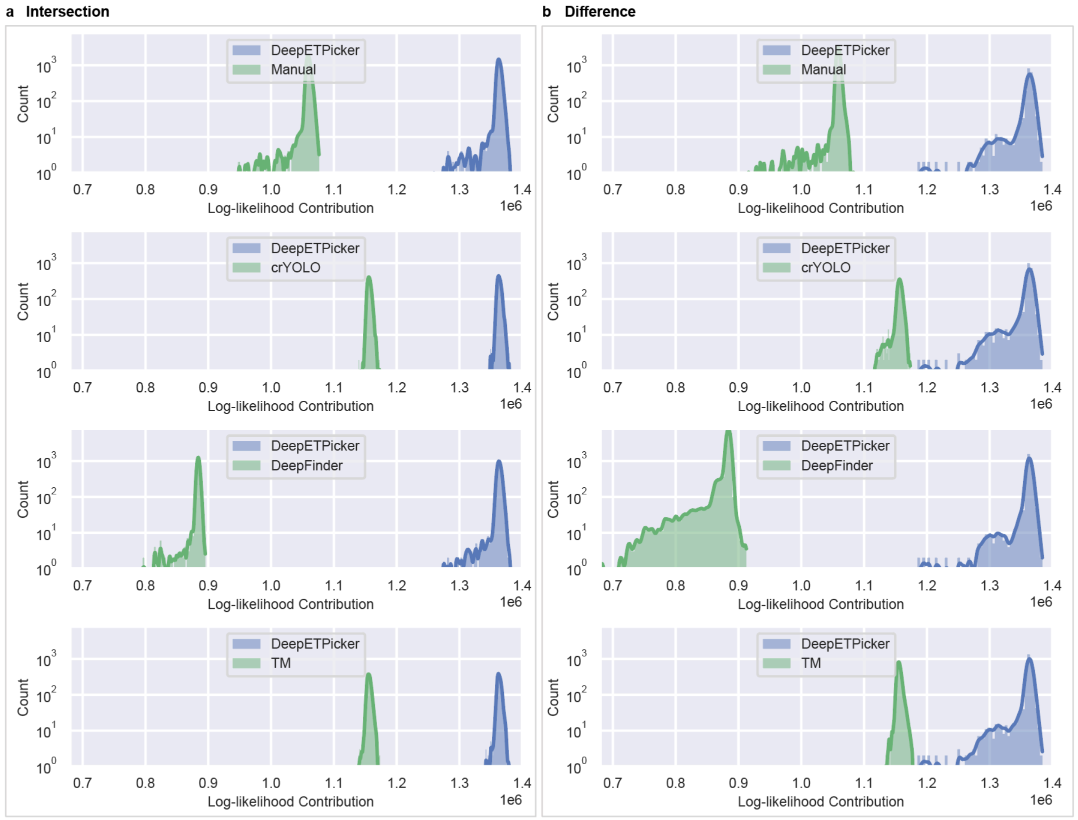

Supplementary Fig. 11 | Log-likelihood contributions of the intersection (a) and difference (b) sets of particles between DeepETPicker and the other three methods on EMPIAR-10499 dataset. The results of DeepETPicker and each of the other three competing methods are shown in blue and green, respectively. Source data are provided as a Source Data file.

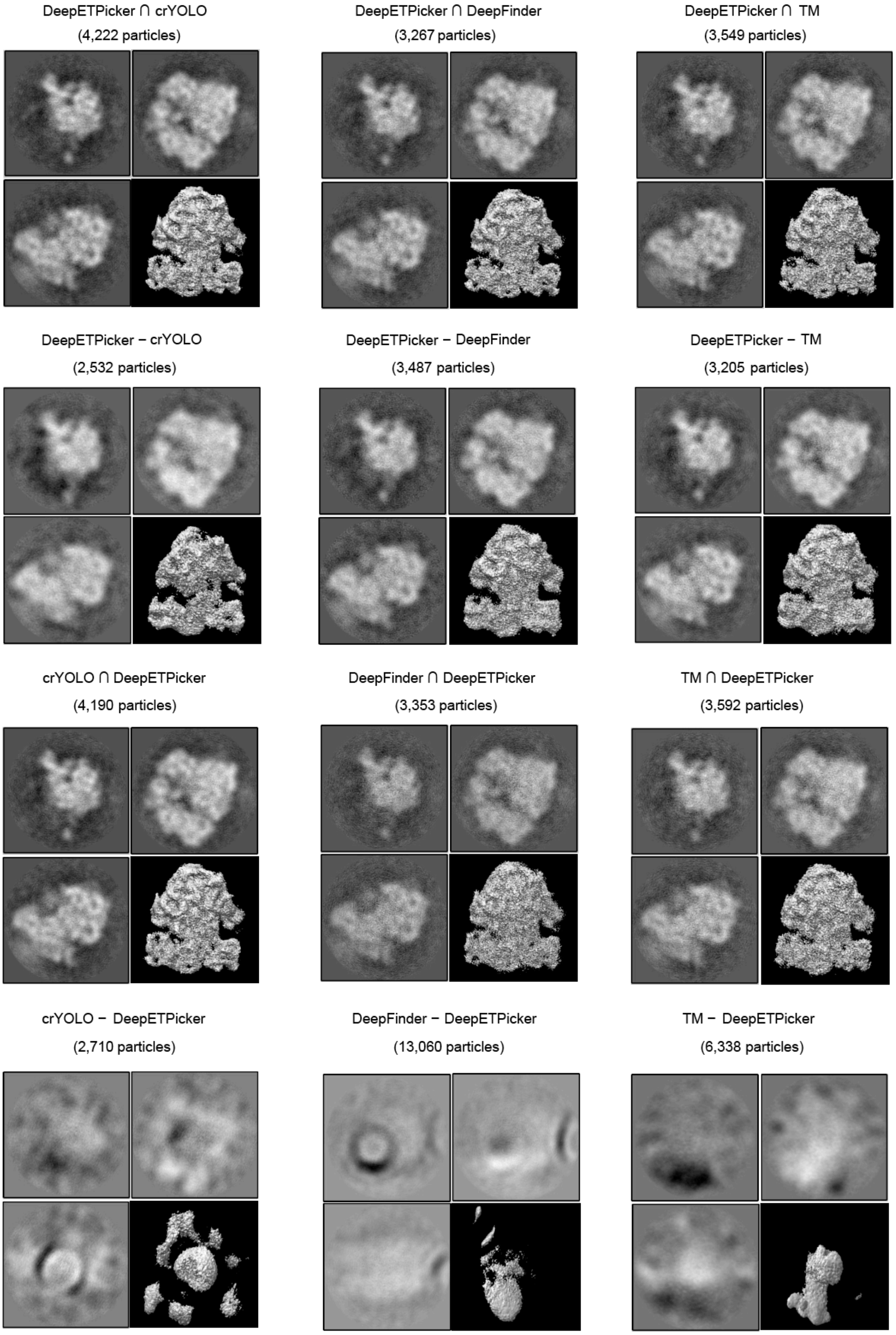

Supplementary Fig. 12 | Comparison of subtomogram averaging for the intersection and difference sets of particles selected by different methods on EMPIAR-10499 dataset of native

***M. pneumoniae* cells.** The four figure panels are the 75th, 95th and 125th sections of the sub-tomogram averaging result and the density map. Source data are provided as a Source Data file.

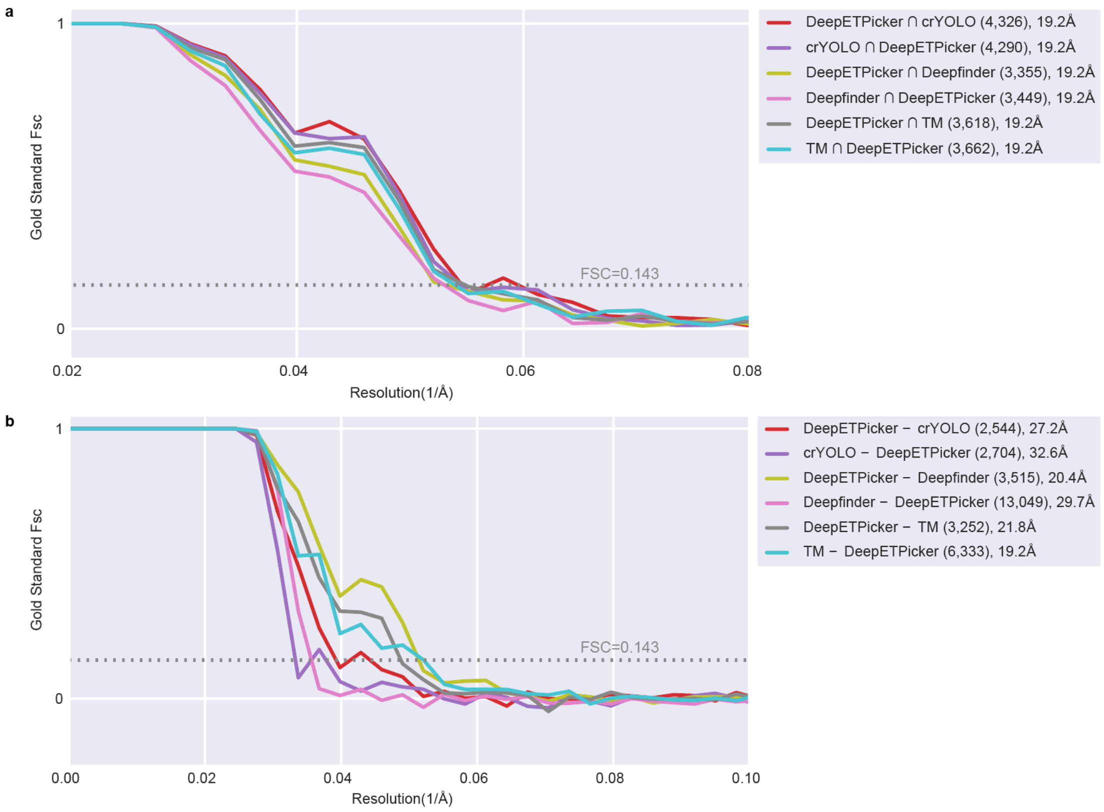

Supplementary Fig. 13 | Comparison of FSC curves achieved by the intersection (a) and difference (b) sets of particles picked by different methods on EMPIAR-10499 dataset of native *M. pneumoniae* cells. Source data are provided as a Source Data file.

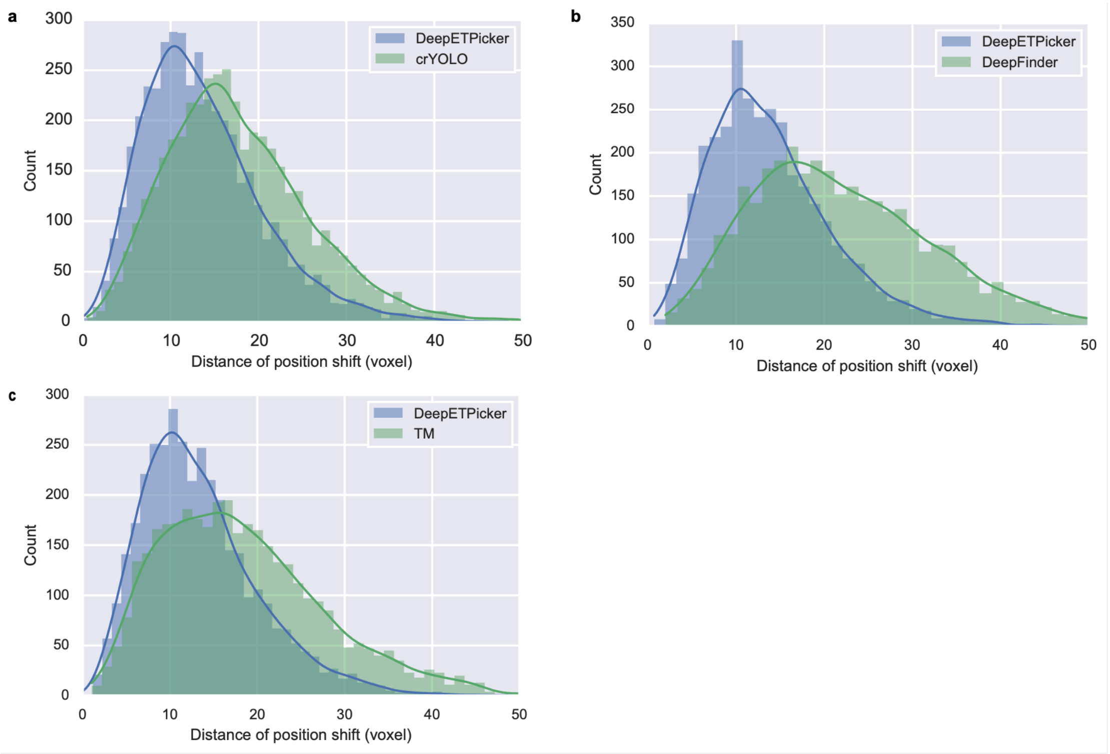

Supplementary Fig. 14 | The two-norm of center shifts for the same particles picked by DeepETPicker and the competing methods on EMPIAR-10499 dataset. (a) DeepETPicker vs crYOLO. (b) DeepETPicker vs DeepFinder. (c) DeepETPicker vs TM. Source data are provided as a Source Data file.

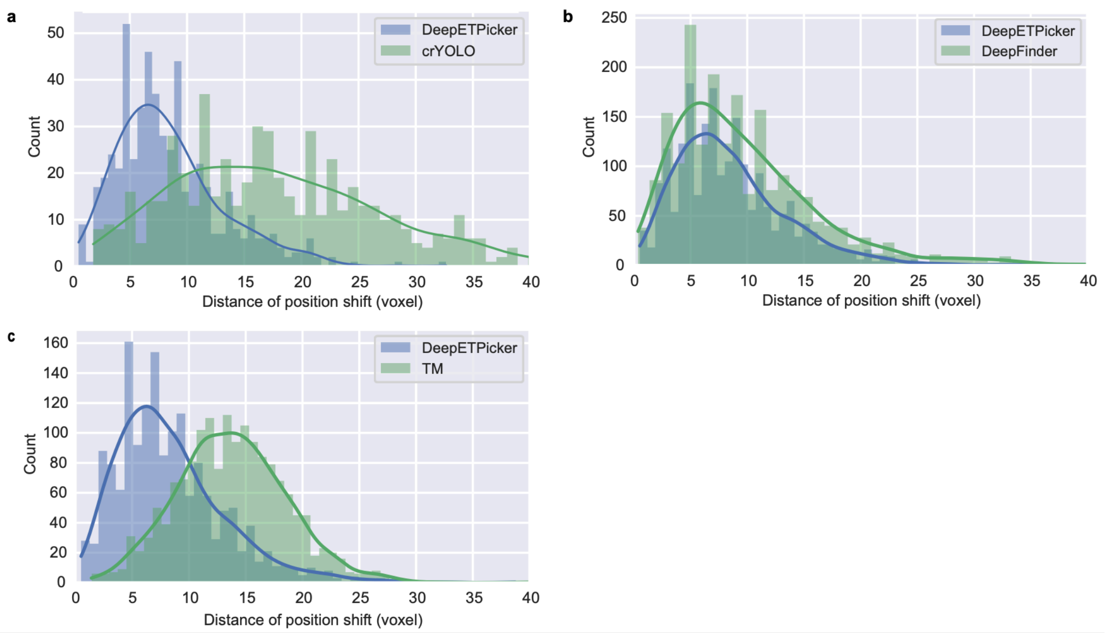

Supplementary Fig. 15 | The two-norm of center shifts for the same particles picked by DeepETPicker and the competing methods on EMPIAR-11125 dataset. (a) DeepETPicker vs crYOLO. (b) DeepETPicker vs DeepFinder. (c) DeepETPicker vs TM. Source data are provided as a Source Data file.

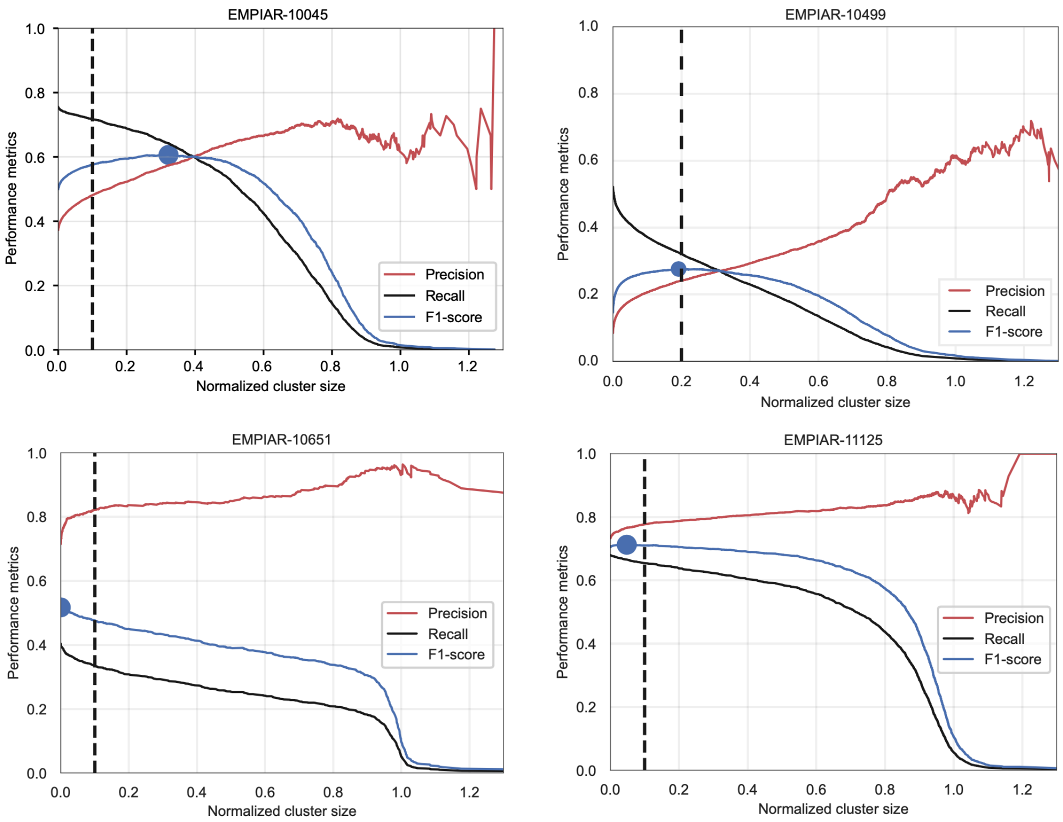

Supplementary Fig. 16. The performance of DeepFinder with different thresholds of cluster size on test set on four experimental datasets. The black vertical dotted line is the threshold corresponding to current result. The blue point corresponds to the maximal of F1-score. Source data are provided as a Source Data file.

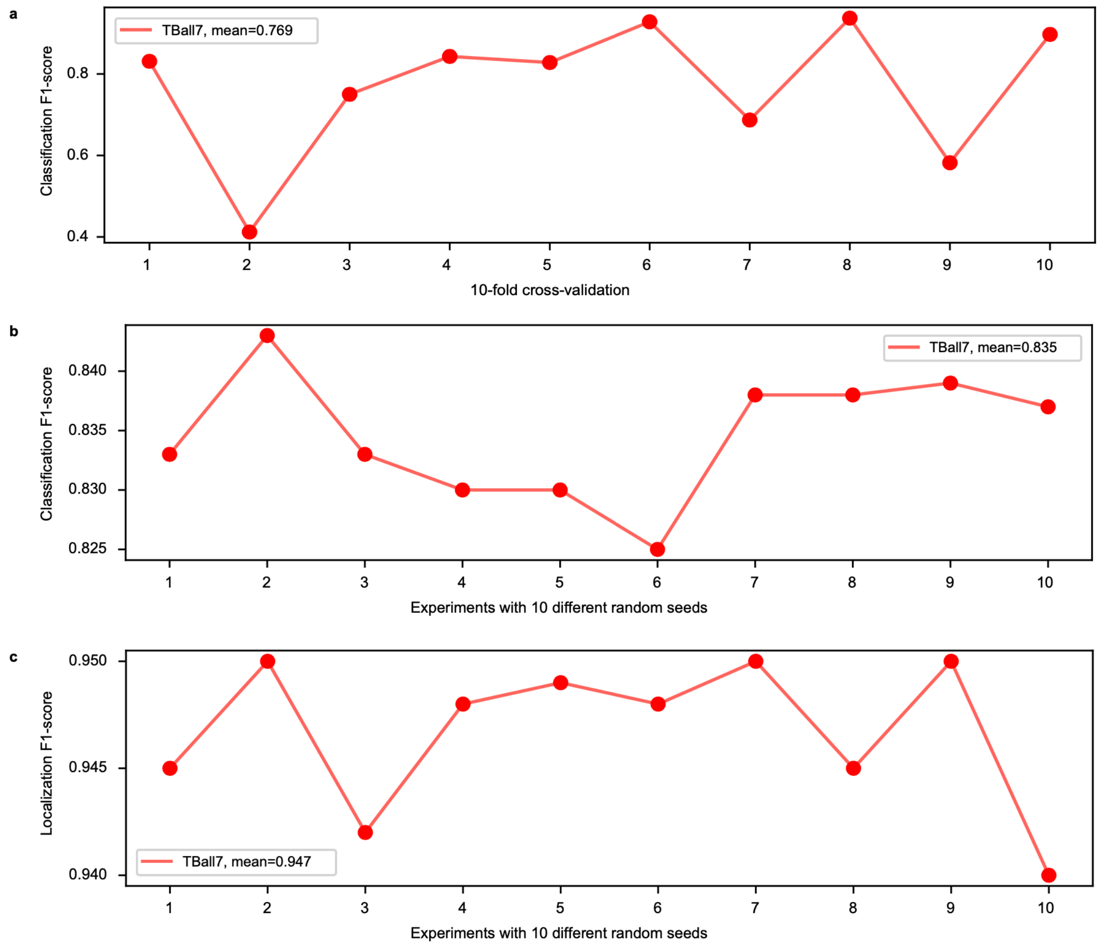

Supplementary Fig. 17. Experiments with multiple training runs on the SHREC2021 dataset. a, 10-fold cross-validation of DeepETPicker trained by TBall-M. **b, c**, Following the same protocol of partitioning the training/validation/test sets as in the SHREC2021 challenge, experiments are performed multiple times by using 10 different random seeds. Source data are provided as a Source Data file.

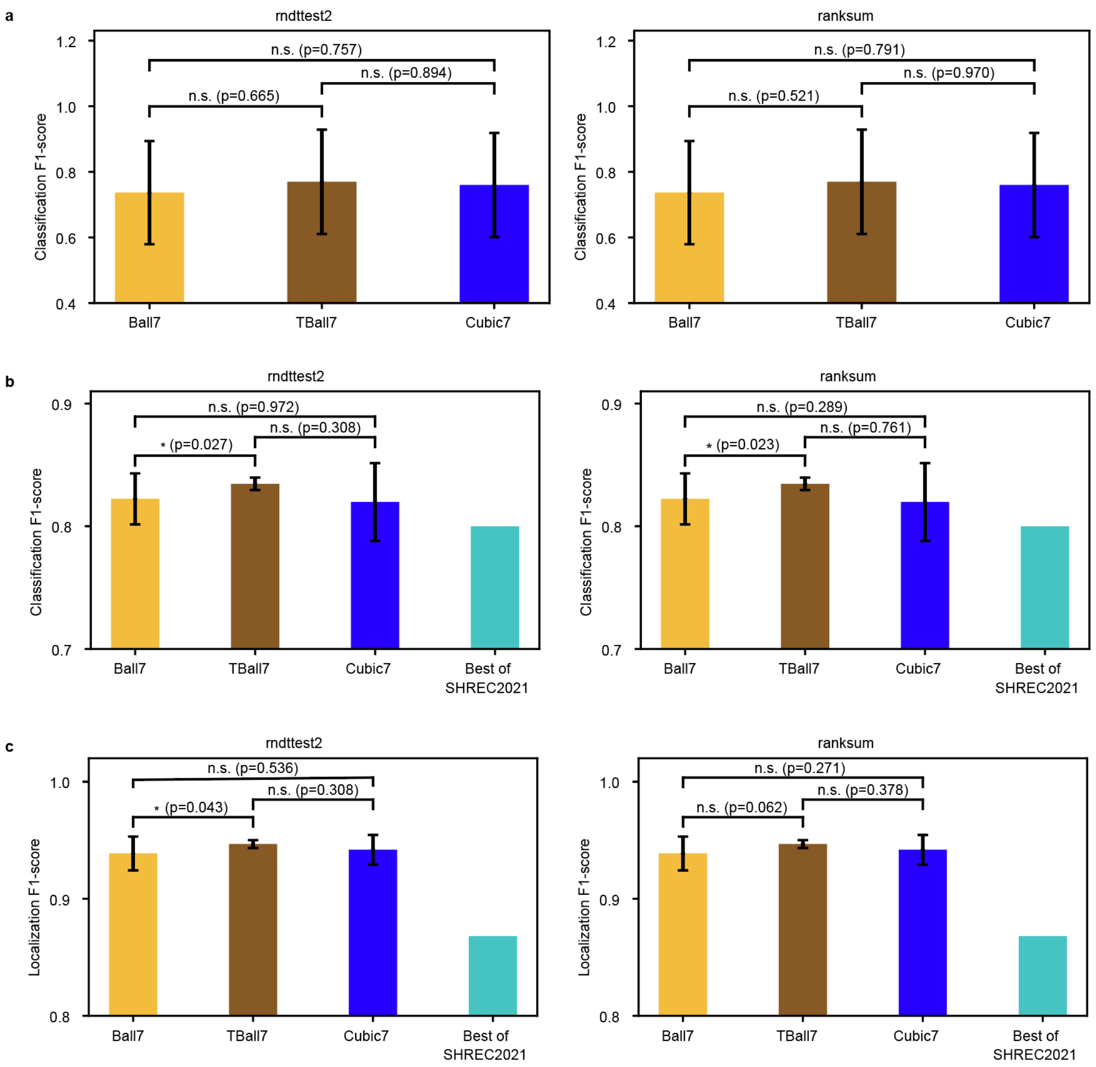

Supplementary Fig. 18. Performance of weak labels with multiple training runs on the SHREC2021 dataset. a, 10-fold cross-validation of DeepETPicker trained by three different simplified masks. **b, c**, Following the same split protocols of training/validation/test set as the challenge, experiments are performed multiple times by using 10 different random seeds. Randomized two sample t-test (rndttest2) as well as nonparametric ranksum test were performed for statistical comparison. Data are presented as mean values, and the error bar denotes the standard deviation of different experiments. 'Best of SHREC2021' denotes the best results of reported methods in SHREC2021 challenges. *: p<0.05; **: p < 0.01; ***: p < 0.001. n.s.: non-significant. Source data are provided as a Source Data file.

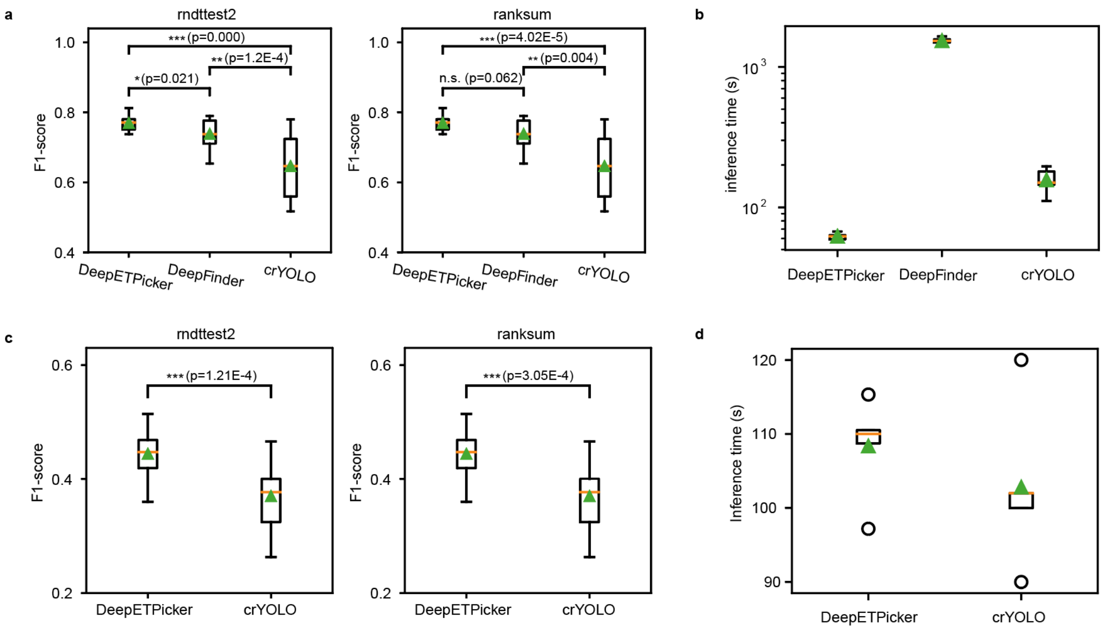

Supplementary Fig. 19. Testing for batch-effect of experimental datasets. a, Boxplot of maximal F1-score of each tomogram for five test results for different methods on EMPIAR-10045 dataset. **b**, The mean inference time for five test results for different methods on EMPIAR-10045 dataset. **c**, Boxplot of maximal F1-score of each tomogram for five test results for different methods on EMPIAR-10499 dataset. **d**, The mean inference time from five test runs for different methods on EMPIAR-10499 dataset. The green upper triangle symbol denotes the mean F1-score of five test results. The inference time is measured by one Nvidia GeForce GTX 2080Ti. Randomized two sample t-test (rndttest2) as well as nonparametric ranksum test were performed for statistical comparison. *: $p<0.05$; **: $p < 0.01$; ***: $p < 0.001$. n.s.: non-significant. Source data are provided as a Source Data file.

C. Supplementary Tables

Supplementary Table 1 | Hyperparameters of DeepETPicker on different datasets. Source data are provided as a Source Data file.

Parameters \ Datasets	SHREC2020	SHREC2021	EMPIAR-10045	EMPIAR-10499	EMPIAR-10651	EMPIAR-11125	Parameter description
t_g	0.368						Threshold for truncated-ball masks
t_{seg}	0.5						Threshold for transforming segmentation maps into binary maps
d	7~19	7~19	25	25	21	13	Diameter of the particles
t_{dist}	$\left\lceil \frac{d}{2} \right\rceil$						Threshold to distinguish whether two adjacent particles are the same particle, where $\lceil \cdot \rceil$ denotes the round-up operation
t_{lm}	0.1						Threshold for determine whether a local maximum is a particle
N	72	72	72	72	72	72	Size of sub-tomograms
pad_size	12	12	12	12	12	12	Padding size for the overlap strategy
lr	0.001	0.001	0.001	0.001	0.001	0.001	Initial learning rate
$batch\_size$	16	16	24	24	24	24	Batch size
max_$epoch$	60	60	60	60	60	60	Total number of training epochs

Supplementary Table 2 | Three types of simplified masks, i.e., truncated-ball masks (TBall-M), cubic masks (Cubic-M) and ball masks (Ball-M), with different diameters. Size Based denotes the diameter of the generated mask is set as proportional to the size of real mask, Const7 and Const9 denote the diameters of the generated masks are set as 7 and 9, respectively. Source data are provided as a Source Data file.

**Simplified Mask**		**1s3x**	**3qm1**	**3gl1**	**3h84**	**2cg9**	**3d2f**	**1u6g**	**3cf3**	**1bxn**	**1qvr**	**4cr2**	**5mrc**	**fiducial**
**TBall-M**	Size Based	7	7	7	7	9	9	9	11	13	13	13	17	7
	Const7							7						
	Const9							9						
**Cubic-M**	Size Based	7	7	7	7	9	9	9	11	13	13	13	17	7
	Const7							7						
	Const9							9						
**Ball-M**	Size Based	7	7	7	7	9	9	9	11	13	13	13	17	7
	Const7							7						
	Const9							9						

Supplementary Table 3 | Localization and classification performance of DeepETPicker trained by different types of simplified masks on the SHREC2021 dataset. Source data are provided as a Source Data file.

		Localization									Classification													
**Mask Type**		**RR**	**TP**	**FP**	**FN**	**MH**	**AD**	**R**	**P**	**F1**	**1s3x**	**3qm**	**3gl1**	**3h84**	**2cg9**	**3d2f**	**1u6g**	**3cf3**	**1bxn**	**1qvr**	**4cr2**	**5mrc**	**fiducial**	**mean F1**
**Real Mask**		1609	1479	70	86	59	1.47	0.94	0.92	0.93	0.39	0.52	0.58	0.81	0.80	0.83	0.79	0.96	0.99	0.95	0.97	0.99	1.00	0.81
**TBall-M**	Size Based	1510	1441	42	124	26	1.16	0.92	0.95	0.94	0.37	0.54	0.60	0.83	0.84	0.89	0.82	0.99	0.99	0.99	1.00	1.00	1.00	0.84
	Const7	1510	1447	43	118	19	1.15	0.92	0.96	0.94	0.41	0.44	0.60	0.88	0.81	0.89	0.81	0.98	1.00	0.99	1.00	1.00	1.00	0.83
	Const9	1508	1446	41	119	18	1.22	0.92	0.96	0.94	0.45	0.54	0.64	0.83	0.81	0.89	0.82	0.97	0.99	0.98	1.00	1.00	1.00	0.84
**Cubic-M**	Size Based	1473	1421	33	144	19	1.22	0.91	0.97	0.93	0.33	0.53	0.60	0.85	0.76	0.87	0.77	0.97	0.98	0.97	1.00	1.00	1.00	0.82
	Const7	1573	1466	82	99	24	1.13	0.93	0.93	0.93	0.46	0.55	0.59	0.83	0.83	0.88	0.83	0.99	1.00	0.96	1.00	1.00	1.00	0.84
	Const9	1543	1438	71	127	31	1.22	0.92	0.93	0.92	0.35	0.47	0.48	0.82	0.77	0.85	0.82	1.00	1.00	0.98	1.00	1.00	1.00	0.81
**Ball-M**	Size Based	1497	1434	43	131	18	1.172	0.91	0.96	0.94	0.46	0.51	0.62	0.75	0.77	0.81	0.81	0.96	0.99	0.97	1.00	1.00	1.00	0.82
	Const7	1284	1202	73	363	9	1.192	0.77	0.94	0.84	0.47	0.46	0.61	0.83	0.81	0.83	0.80	0.97	0.99	0.97	0.00	0.00	1.00	0.67
	Const9	1474	1418	43	147	11	1.214	0.90	0.96	0.93	0.36	0.53	0.63	0.88	0.81	0.87	0.80	0.99	1.00	0.98	1.00	1.00	1.00	0.84

Supplementary Table 4 | Comparison of DeepETPicker versus competing methods in localization performance. The methods such as URFinder, DeepFinder, U-CLSTM, MC-DS-Net, YOPO, TM-F and TM are reported in the SHREC2021 challenge[2]. RR: detected number of particles; TP: true positive; FP: false positive, FN: false negative, MH: multiple hits, AD: average Euclidean distance from predicted particle center in voxels; Recall: uniquely selected true locations divided by actual number of particles in the test tomogram; Precision: uniquely selected true locations divided by RR; Miss rate: percentage of results that yield negative results; F1 Score: harmonic average of the precision and recall. The best results in each column are highlighted in bold. ↑ indicates that the higher the better, ↓ indicates that the lower the better. Source data are provided as a Source Data file.

Methods	RR	TP ↑	FP ↓	FN ↓	MH ↓	AD ↓	Recall ↑	Precision ↑	Miss rate ↓	F1 Score ↑
**URFinder**	1969	1298	377	267	149	1.84	0.826	0.659	0.174	0.733
**YOPO**	1627	1224	232	341	**14**	1.66	0.720	0.752	0.221	0.765
**CFN**	1765	1364	239	201	20	1.52	0.868	0.773	0.132	0.818
**U-CLSTM**	1460	1253	49	312	44	2.13	0.798	0.858	0.202	0.827
**MC DS Net**	1760	1415	239	150	56	1.59	0.901	0.804	0.099	0.850
**DeepFinder**	1567	1362	64	203	20	2.22	0.867	0.869	0.133	0.868
**DeepETPicker**	1510	**1447**	**43**	**118**	19	**1.15**	**0.921**	**0.958**	**0.079**	**0.939**

Supplementary Table 5 | Comparison of DeepETPicker versus competing methods in classification performance measured in F1-score on specific particle classes. The competing methods such as URFinder, DeepFinder, U-CLSTM, MC-DS-Net, YOPO, TM-F and TM are reported in SHREC2021 challenge[2]. The best results in each column are highlighted in bold. Source data are provided as a Source Data file.

Methods	1s3x	3qm1	3gl1	3h84	2cg9	3d2f	1u6g	3cf3	1bxn	1qvr	4cr2	5mrc	fiducial	mean F1
**URFinder**	**0.00**	**0.42**	**0.45**	**0.60**	**0.54**	**0.67**	**0.67**	**0.87**	**0.97**	**0.86**	**0.93**	**0.95**	**0.43**	**0.64**
**YOPO**	0.20	0.15	0.47	0.60	0.63	0.63	0.61	0.88	0.94	0.92	0.98	0.97	0.95	0.69
**U-CLSTM**	0.28	0.42	0.39	0.56	0.51	0.65	0.57	0.95	0.99	0.90	0.99	**1.00**	**1.00**	0.71
**DeepFinder**	0.40	0.48	0.52	0.70	0.72	0.77	0.74	0.96	0.99	0.95	0.97	1.00	**1.00**	0.78
**CFN**	0.25	**0.51**	**0.61**	0.77	0.71	0.76	0.73	0.97	**1.00**	0.97	1.00	**1.00**	**1.00**	0.79
**MC DS Net**	0.32	0.49	0.60	0.78	0.78	0.79	0.80	0.96	0.99	0.93	0.98	**1.00**	**1.00**	0.80
**DeepETPicker**	**0.41**	0.44	0.60	**0.88**	**0.81**	**0.89**	**0.81**	**0.98**	**1.00**	**0.99**	**1.00**	**1.00**	**1.00**	**0.83**

Supplementary Table 6 | Reported computing time in training and inference stages for processing one SHREC2021 tomogram of size $200 \times 512 \times 512$. Calculation of estimated speedup ratios of DeepETPicker in the inference stage is based on the assumption that the calculation is mainly completed by the GPU. Source data are provided as a Source Data file.

Methods	Training stage	Inference stage	Hardware	FP32 TFLOPS	Estimated speedup ratio of DeepETPicker in inference stage
URFinder	300h	2h6m	Nvidia Quodro RTX 8000 GPU (2×)	16.3 x 2	654.42
DeepFinder	50h	20m	Nvidia M40 (1×)	6.83	21.76
U-CLSTM	120h	15m	Nvidia Quodro RTX-5000 GPU (1×)	11.2	26.77
MC DS Net	22h	5m	Nvidia GeForce RTX 3090 GPU (1×)	35.58	28.34
YOPO	8h	40m	Nvidia GeForce Titan X GPU (1×)	6.69	42.63
CFN	96h	-	Nvidia GeForce RTX 3090 GPU (2×)	35.58 x 2	-
TM-F/TM GPU	N/A	4h26m	Nvidia GeForce GTX 1080Ti (1×)	11.34	480.58
DeepETPicker	17h	28s	Nvidia GeForce GTX 2080Ti (1×)	13.45	1.00

Supplementary Table 7 | Relationship between standard deviation of Gaussian kernels and SNR levels of the SHREC2021 dataset. Source data are provided as a Source Data file.

**Standard deviation of Gaussian kernel**	**SNR level of SHREC2021 datasets**
0	0.127-0.587
0.5	0.116-0.535
1.1	0.101-0.463
1.5	0.077-0.348
2	0.056-0.254
3	0.039-0.171
5	0.026-0.110

Supplementary Table 8 | Ablation Study of DeepETPicker. RC=Residual Connection, CC=Coord Conv, IP=Image Pyramid, DA=Data Augmentation, DD=De-Duplication, OT=Overlap-tile Strategy. Source data are provided as a Source Data file.

Ablation Study						Localization									Classification													
**RC**	**CC**	**IP**	**DA**	**DD**	**OT**	**RR**	**TP**	**FP**	**FN**	**MH**	**AD**	**R**	**P**	**F1**	**1s3x**	**3qm**	**3gl1**	**3h84**	**2cg9**	**3d2f**	**1u6g**	**3cf3**	**1bxn**	**1qvr**	**4cr2**	**5mrc**	**fiducial**	**mean F1**
						1353	1221	17	344	106	1.40	0.78	0.90	0.84	0.20	0.36	0.37	0.57	0.59	0.67	0.58	0.85	0.98	0.87	0.98	0.97	1.00	0.69
✓						1295	1197	21	368	71	1.38	0.76	0.92	0.84	0.21	0.33	0.39	0.62	0.61	0.67	0.61	0.86	0.97	0.89	0.97	0.97	1.00	0.70
✓	✓	✓				1528	1286	28	279	184	1.49	0.82	0.84	0.83	0.36	0.37	0.39	0.59	0.61	0.68	0.66	0.85	0.97	0.85	0.97	0.95	1.00	0.71
✓	✓	✓	✓			1424	1314	35	251	72	1.27	0.84	0.92	0.88	0.38	0.42	0.56	0.77	0.72	0.83	0.77	0.93	1.00	0.92	0.98	0.99	1.00	0.79
✓	✓	✓	✓	✓		1376	1313	33	252	28	1.27	0.84	0.95	0.89	0.37	0.42	0.54	0.77	0.73	0.83	0.77	0.95	1.00	0.93	0.98	0.99	1.00	0.79
✓	✓	✓	✓	✓	✓	1510	1447	43	118	19	1.15	0.92	0.96	0.94	0.41	0.44	0.60	0.88	0.81	0.89	0.81	0.98	1.00	0.99	1.00	1.00	1.00	0.83

Supplementary Table 9 | Comparison of DeepETPicker versus competing methods in global resolution achieved by the intersection and difference sets of particles selected by different methods on EMPIAR-10045 dataset of S. cerevisiae 80S ribosome. RH resolution is the theoretical resolution estimated based on the Rosenthal and Henderson B-factor plot (RH plot)[1]. Source data are provided as a Source Data file.

Method	No of particles for 3D reconstruction	Resolution (Å)	RH Resolution (Å)	Method	No of particles for 3D reconstruction	Resolution (Å)	RH Resolution (Å)
DeepETPicker ∩ reported result	2533	15.5	15.7	reported result ∩ DeepETPicker	2571	15.6	-
DeepETPicker − reported result	1284	18.1	17.8	reported result − DeepETPicker	138	31.2	-
DeepETPicker ∩ crYOLO	2542	15.0	15.7	crYOLO ∩ DeepETPicker	2507	15.5	16.0
DeepETPicker − crYOLO	1275	18.9	17.8	crYOLO − DeepETPicker	300	31.0	24.7
DeepETPicker ∩ DeepFinder	3038	15.0	15.3	DeepFinder ∩ DeepETPicker	2996	15.0	16.4
DeepETPicker − DeepFinder	779	20.7	19.9	DeepFinder − DeepETPicker	1433	28.9	18.8
DeepETPicker ∩ TM	3613	15.0	14.9	TM ∩ DeepETPicker	3555	15.0	16.2
DeepETPicker − TM	204	33.4	35.6	TM − DeepETPicker	2742	28.9	17.0

Supplementary Table 10 | Comparison of DeepETPicker versus competing methods in global resolution achieved by the intersection and difference sets of particles selected by different methods on EMPIAR-10499 dataset of native M. pneumoniae cells. RH resolution is the theoretical resolution estimated based on the Rosenthal and Henderson B-factor plot (RH plot)[1]. Source data are provided as a Source Data file.

Method	No of particles picked & selected for 3D reconstruction	Resolution (Å)	RH Resolution (Å)	Method	No of particles picked & selected for 3D reconstruction	Resolution (Å)	RH Resolution (Å)
DeepETPicker ∩ crYOLO	4222	19.2	19.3	crYOLO ∩ DeepETPicker	4190	19.2	20.3
DeepETPicker − crYOLO	2532	27.2	20.6	crYOLO − DeepETPicker	2710	32.6	21.4
DeepETPicker ∩ DeepFinder	3267	19.2	20.0	DeepFinder ∩ DeepETPicker	3353	19.2	34.0
DeepETPicker − DeepFinder	3487	20.4	19.8	DeepFinder − DeepETPicker	13060	29.7	29.5
DeepETPicker ∩ TM	3549	19.2	19.8	TM ∩ DeepETPicker	3592	19.2	22.9
DeepETPicker − TM	3205	21.8	20.0	TM − DeepETPicker	6338	19.2	20.9

Supplementary Table 11 | Influence of t_{seg} on classification performance of DeepETPicker trained by different types of masks on the SHREC2021 dataset. Source data are provided as a Source Data file.

Label Type	t_{seg}								
	0.1	0.2	0.3	0.4	0.5	0.6	0.7	0.8	0.9
Real Mask	0.817	0.816	0.816	0.815	0.815	0.815	0.815	0.815	0.815
TBall-M, d=9	0.830	0.830	0.830	0.830	0.830	0.831	0.831	0.830	0.830
Cubic-M, d=9	0.827	0.827	0.828	0.828	0.828	0.828	0.828	0.827	0.828
Ball-M, d=9	0.829	0.828	0.828	0.829	0.829	0.829	0.829	0.829	0.828

Supplementary Table 12 | An ablation study for coordinated convolution and image pyramid inputs on the SHREC2021 dataset. CC denotes coordinate convolution, and IP denotes image pyramid inputs. Tomograms 0 to 2 are used for training of DeepETPicker, tomogram 8 is used for validation and tomogram 9 is used for testing. In the ablation study, *pad_size* is set as 0, and data augmentation is not used. Source data are provided as a Source Data file.

Ablation Study		Mean F1-score of classification				
CC	IP	Tiny	Small	Medium	Large	Total
		0.227	0.488	0.830	0.922	0.612
√		0.234	0.500	0.871	0.915	0.627
	√	0.253	0.485	0.868	0.928	0.627
√	√	0.308	0.525	0.873	0.930	0.654

Supplementary Table 13 | An ablation study for channels of 3D-ResUNet on the SHREC2021 dataset. Tomograms 0 to 7 are used for training of DeepETPicker, tomogram 8 is used for validation and tomogram 9 is used for testing. Source data are provided as a Source Data file.

Channels [c1, c2, c3, c4]	Model size	Localization performance				Classification Performance
		AD	Recall	Precision	F1-score	Mean F1-score
[24, 48, 72, 108]	28M	1.18	0.933	0.961	0.947	0.835
[8, 16, 24, 36]	3.4M	1.217	0.927	0.944	0.935	0.829
DeepFinder	11M	2.22	0.867	0.869	0.868	0.784

Supplementary Table 14. An ablation study for loss functions on the SHREC2021 dataset. Truncated-Ball masks with constant diameter of 9 is used. Tomograms 0 to 2 are used for training of DeepETPicker, tomogram 8 is used for validation and tomogram 9 is used for testing. Source data are provided as a Source Data file.

Loss function	Localization performance				Classification
	AD	Recall	Precision	F1-score	mean F1-score
Dice	1.34	0.902	0.971	0.935	0.786
MSE	1.417	0.908	0.978	0.942	0.773
Focal	1.373	0.911	0.977	0.943	0.759
IoU	1.304	0.881	0.985	0.93	0.767

Supplementary Table 15. An ablation study for different transformations on the SHREC2021 dataset. Tomograms 0 to 2 are used for training of DeepETPicker, tomogram 8 is used for validation and tomogram 9 is used for testing. Source data are provided as a Source Data file.

Mirror Transformation	Spatial Transformation	Localization performance				Classification
		AD	Recall	Precision	F1-score	mean F1-score
		1.513	0.859	0.947	0.901	0.691
√		1.372	0.891	0.932	0.911	0.774
	√	1.469	0.878	0.968	0.921	0.711
√	√	1.34	0.902	0.971	0.935	0.786

Supplementary Table 16. The classification F1-score of DeepFinder with and without volume threshold on test set of four experimental datasets. Source data are provided as a Source Data file.

**Dataset name**	**Without volume threshold (default result of DeepFinder)**	**With volume threshold**
**EMPIAR-10045**	0.500	0.576
**EMPIAR-10499**	0.146	0.276
**EMPIAR-10651**	0.510	0.468
**EMPIAR-11125**	0.705	0.711

Supplementary Table 17. Cross-correlation threshold of TM for different datasets. Source data are provided as a Source Data file.

EMPIAR-10045		EMPIAR-10499	
IS002_291013_005	0.182	TS_77	0.060
IS002_291013_006	0.188	TS_78	0.060
IS002_291013_007	0.163	TS_79	0.062
IS002_291013_008	0.150	TS_80	0.065
IS002_291013_009	0.160	TS_81	0.050
IS002_291013_010	0.170	TS_82	0.053
IS002_291013_011	0.157	TS_84	0.057
		TS_85	0.059
		TS_87	0.061
		TS_88	0.063
**EMPIAR-10651**		**EMPIAR-11125**	
k2dft20s_14apra0006	0.440	CB_02	0.250
k2dft20s_14apra0011	0.440	CB_29	0.240
k2dft20s_14apra0023	0.440	CB_59	0.260

Supplementary Table 18. Training, validation, testing set of different deep-learning based methods for experimental datasets. Source data are provided as a Source Data file.

Dataset name	Number of tomograms (index)	Manually labelled particles from all tomograms	Number of particles for training	Number of particles for validation	Number of particles for testing (excluded training and validation particles)	Particle picking methods
**EMPIAR-10045**	7 (tomo0-tomo6)	3120	135 from tomo0	15 from tomo1	2970 particles from tomo0-tomo6	DeepETPicker<br>crYOLO<br>DeepFinder
**EMPIAR-10499**	10 (tomo0-tomo9)	12624	106 from tomo5	11 from tomo4	11921 particles from tomo0-tomo9	DeepETPicker<br>crYOLO
			650 from tomo5	53 from tomo4		DeepFinder
**EMPIAR-10651**	3 (tomo0-tomo2)	1340	128 from tomo2	14 from tomo1	1198 from tomo0-tomo2	DeepETPicker<br>crYOLO<br>DeepFinder
**EMPIAR-11125**	3 (tomo0-tomo2)	2972	514 from tomo0	57 from tomo1	2401 from tomo0-tmo2	DeepETPicker<br>crYOLO<br>DeepFinder

Supplementary Table 19. Tomogram names and their abbreviation for experimental datasets. Source data are provided as a Source Data file.

Dataset	Original names of tomograms	Shortened names of tomograms
**EMPIAR-10045**	IS002_291013_005	tomo0
	IS002_291013_006	tomo1
	IS002_291013_007	tomo2
	IS002_291013_008	tomo3
	IS002_291013_009	tomo4
	IS002_291013_010	tomo5
	IS002_291013_011	tomo6
**EMPIAR-10651**	k2dft20s_14apra0006	tomo0
	k2dft20s_14apra0011	tomo1
	k2dft20s_14apra0023	tomo2
**EMPIAR-10499**	TS_77	tomo0
	TS_78	tomo1
	TS_79	tomo2
	TS_80	tomo3
	TS_81	tomo4
	TS_82	tomo5
	TS_84	tomo6
	TS_85	tomo7
	TS_87	tomo8
	TS_88	tomo9
EMPIAR-11125	CB_02	tomo0
	CB_29	tomo1
	CB_59	tomo2

Supplementary Table 20. Mean and standard deviation of all experiments performed multiple times by using 10 different random seeds. Source data are provided as a Source Data file.

Metrics		TBall7	Cubic7	Ball7
**Classification F1-score**	**Mean**	0.835	0.820	0.822
	**Standard deviation**	0.005	0.032	0.021
**Localization F1-score**	**Mean**	0.947	0.942	0.939
	**Standard deviation**	0.003	0.013	0.014

Supplementary Table 21. Training, validation, testing set of batch-effect experiments on two experimental datasets. Source data are provided as a Source Data file.

Dataset name	Number of tomograms (index)	Train set1	Train set2	Train set3	Train set4	Train set5	Validation set	Test set
**EMPIAR-10045**	7 (tomo0-tomo6)	135 from tomo0	135 from tomo0	135 from tomo1	135 from tomo1	135 from tomo3	15 from tomo2	1391 from tomo4-tomo6
**EMPIAR-10499**	10 (tomo0-tomo9)	106 from tomo0	106 from tomo1	106 from tomo2	106 from tomo3	106 from tomo5	11 from tomo4	6429 from tomo6-tomo9

Supplementary References

1 Rosenthal, P. B. & Henderson, R. Optimal determination of particle orientation, absolute hand, and contrast loss in single-particle electron cryomicroscopy. *Journal of Molecular Biology* 333, 721-745 (2003).

2 Gubins, I. *et al.* SHREC 2021: classification in cryo-electron tomograms. in *Eurographics Workshop on 3D Object Retrieval.* (eds Silvia Biasotti *et al.*), The Eurographics Association, (2021).
