# Supplementary material for: DeepETPicker: Fast and accurate 3D particle picking for cryo-electron tomography using weakly supervised deep learning
Source: Nat Commun. 2024 Mar 7;15:2090. doi: 10.1038/s41467-024-46041-0 (PMC11258139; doi:10.1038/s41467-024-46041-0)
Supplement: Supplementary file 3 — Description of Additional Supplementary Files [file 41467_2024_46041_MOESM3_ESM.pdf]

Description of Additional Supplementary Files:

Supplementary Movie 1: Comparison of particles picked by DeepETPicker versus the other four competing methods (reported result: the method reported in the original article (Bharat & Scheres, Nature Protocols, 11:2054-2065, 2016), crYOLO, template matching, and Deepfinder) on the EMPIAR-10045 experimental dataset. Different colors show the same and different particles detected. Intersection sets of particles picked by DeepETPicker and the other competing method are shown blue. Difference sets of particles picked by DeepETPicker and the other competing method are shown in red and cyan, respectively.

Supplementary Movie 2: Comparison of particles picked by DeepETPicker versus the other three competing methods (crYOLO, Deepfinder, and template matching) on the EMPIAR-10499 experimental dataset. Different colors show the same and different particles detected. Intersection sets of particles picked by DeepETPicker and the other competing method are shown in blue. Difference sets of particles picked by DeepETPicker and the other competing method are shown in red and cyan, respectively.
