# Supplementary material for: DeepETPicker: Fast and accurate 3D particle picking for cryo-electron tomography using weakly supervised deep learning
Source: Nat Commun. 2024 Mar 7;15:2090. doi: 10.1038/s41467-024-46041-0 (PMC11258139; doi:10.1038/s41467-024-46041-0)
Supplement: Supplementary file 6 — Reporting Summary [file 41467_2024_46041_MOESM6_ESM.pdf]

nature portfolio

Corresponding author(s): Ge Yang, Fei Sun

Last updated by author(s): Jan 4, 2024

Reporting Summary

Nature Portfolio wishes to improve the reproducibility of the work that we publish. This form provides structure for consistency and transparency in reporting. For further information on Nature Portfolio policies, see our Editorial Policies and the Editorial Policy Checklist.

Statistics

For all statistical analyses, confirm that the following items are present in the figure legend, table legend, main text, or Methods section.

n/a	Confirmed	
☐	☒	The exact sample size (*n*) for each experimental group/condition, given as a discrete number and unit of measurement
☐	☒	A statement on whether measurements were taken from distinct samples or whether the same sample was measured repeatedly
☐	☒	The statistical test(s) used AND whether they are one- or two-sided<br>*Only common tests should be described solely by name; describe more complex techniques in the Methods section.*
☒	☐	A description of all covariates tested
☒	☐	A description of any assumptions or corrections, such as tests of normality and adjustment for multiple comparisons
☐	☒	A full description of the statistical parameters including central tendency (e.g. means) or other basic estimates (e.g. regression coefficient) AND variation (e.g. standard deviation) or associated estimates of uncertainty (e.g. confidence intervals)
☐	☒	For null hypothesis testing, the test statistic (e.g. *F*, *t*, *r*) with confidence intervals, effect sizes, degrees of freedom and *P* value noted<br>*Give P values as exact values whenever suitable.*
☒	☐	For Bayesian analysis, information on the choice of priors and Markov chain Monte Carlo settings
☒	☐	For hierarchical and complex designs, identification of the appropriate level for tests and full reporting of outcomes
☒	☐	Estimates of effect sizes (e.g. Cohen's *d*, Pearson's *r*), indicating how they were calculated

Our web collection on statistics for biologists contains articles on many of the points above.

Software and code

Policy information about availability of computer code

Data collection	All data used in the study are publicly available. No additional data collection was performed in this study.
Data analysis	DeepETPicker has been implemented using Python 3.8.3 and is mainly based on the Pytorch and PyQT packages. It has been tested on Linux (Ubuntu 18.04 and CentOS) (see https://github.com/cbmi-group/DeepETPicker).<br><br>The software requires NVIDIA GPU for model training and testing. The present code has been tested on NVIDIA GeForce RTX 2080 Ti and NVIDIA GeForce RTX 1080 Ti GPUs. For running on other GPUs, some parameter values need to be changed to adapt to available memory.<br><br>Versions of software used in this study: IMOD v4.9.12, Warp v1.0.9, Relion v2.1.0, Dynamo v1.1.319_MCR-9.2.0_GLNXA64_withMCR, crYOLO v1.8.0b10, DeepFinder 20200129, DeepETPicker: https://github.com/cbmi-group/DeepETPicker, Python v3.8.3, Pytorch v1.7.1, PyQT v5.15.4, numpy v1.19.2.

For manuscripts utilizing custom algorithms or software that are central to the research but not yet described in published literature, software must be made available to editors and reviewers. We strongly encourage code deposition in a community repository (e.g. GitHub). See the Nature Portfolio guidelines for submitting code & software for further information.

Data

Policy information about availability of data

All manuscripts must include a data availability statement. This statement should provide the following information, where applicable:

- Accession codes, unique identifiers, or web links for publicly available datasets
- A description of any restrictions on data availability
- For clinical datasets or third party data, please ensure that the statement adheres to our policy

The simulated tomogram dataset SHREC2020 is available from the website of the SHREC2020 challenge (https://www.shrec.net/cryo-et/2020/).

The simulated tomogram dataset SHREC2021 is available from the website of the SHREC2021 challenge (https://www2.projects.science.uu.nl/shrec/cryo-et/).

The experimental tomogram dataset of purified S. cerevisiae 80S ribosomes is available from EMPIAR under accession number EMPIAR-10045 (https://www.ebi.ac.uk/empiar/EMPIAR-10045).

The experimental tomogram dataset of purified T20S proteasomes is available from EMPIAR under accession number EMPIAR-10651 (https://www.ebi.ac.uk/empiar/EMPIAR-10651)

The experimental tomogram dataset of M. pneumoniae cells is available from EMPIAR under accession number EMPIAR-10499 (https://www.ebi.ac.uk/empiar/EMPIAR-10499).

The experimental tomogram dataset of H. neapolitanus alpha-carboxysomes in situ is available from EMPIAR under accession number EMPIAR-11125 (https://www.ebi.ac.uk/empiar/EMPIAR-11125)

For template matching, reference maps are available from EMDB entry (EMD-0732, EMD-12531, EMD-21562, and EMD-27654).
Source data are provided with this paper.

Research involving human participants, their data, or biological material

Policy information about studies with human participants or human data. See also policy information about sex, gender (identity/presentation), and sexual orientation and race, ethnicity and racism.

Reporting on sex and gender	N/A
Reporting on race, ethnicity, or other socially relevant groupings	N/A
Population characteristics	N/A
Recruitment	N/A
Ethics oversight	N/A

Note that full information on the approval of the study protocol must also be provided in the manuscript.

Field-specific reporting

Please select the one below that is the best fit for your research. If you are not sure, read the appropriate sections before making your selection.

☒ Life sciences ☐ Behavioural & social sciences ☐ Ecological, evolutionary & environmental sciences

For a reference copy of the document with all sections, see nature.com/documents/nr-reporting-summary-flat.pdf

Life sciences study design

All studies must disclose on these points even when the disclosure is negative.

Sample size	Sample sizes were chosen based on experimental validation. No statistical method was used to predetermine sample sizes. DeepETPicker is deep-learning-based software, in terms of hyperparameters such as learning rate, batch size, we finetuned them until DeepETPicker pick particles stably. All hyperparamters for different experiments are provided.
Data exclusions	No data were excluded in the experiments.
Replication	We performed 10 independent random replicates of the training and testing procedures on the simulated datasets . The results are reproducible.
Randomization	Randomization is applied in the initialization of model weights and batch generation during the training procedure (based on stochastic gradient descent). The results of each training are therefore slightly different. However, for large datasets and enough training times, model

	performance after different training cycles is consistent.
Blinding	The purpose of this study is to develop particle picking software for structural biology research. Because no group allocation was performed, blinding was not necessary or possible.

Reporting for specific materials, systems and methods

We require information from authors about some types of materials, experimental systems and methods used in many studies. Here, indicate whether each material, system or method listed is relevant to your study. If you are not sure if a list item applies to your research, read the appropriate section before selecting a response.

Materials & experimental systems

n/a	Involved in the study	
☒	☐	Antibodies
☒	☐	Eukaryotic cell lines
☒	☐	Palaeontology and archaeology
☒	☐	Animals and other organisms
☒	☐	Clinical data
☒	☐	Dual use research of concern
☒	☐	Plants

Methods

n/a	Involved in the study	
☒	☐	ChIP-seq
☒	☐	Flow cytometry
☒	☐	MRI-based neuroimaging
